# Pulmonary sclerosing pneumocytoma and mortality risk

**DOI:** 10.1186/s12890-022-02199-1

**Published:** 2022-11-07

**Authors:** So Jeong Kim, Hye-Rin Kang, Choon Geun Lee, Seung Ho Choi, Yeon Wook Kim, Hyun Woo Lee, Chang-Hoon Lee

**Affiliations:** 1 Division of Pulmonary and Critical Care Medicine, Department of Internal Medicine, Seoul National University Hospital, 101 Daehak-ro, Jongno-gu, Seoul, 03080 Republic of Korea; 2 Department of Pulmonology and Allergy, Hallym University Dongtan Sacred Heart Hospital, Hwaseong, South Korea; 3 Division of Pulmonary, Allergy and Critical Care Medicine, Department of Internal Medicine, Veterans Health Service Medical Center, Seoul, Republic of Korea; 4 Hospital Medicine Center, Seoul National University Hospital, Seoul, South Korea; 5 Department of Internal Medicine, Healthcare Research Institute, Healthcare System Gangnam Center, Seoul National University Hospital, Seoul, South Korea; 6 Division of Pulmonary and Critical Care Medicine, Department of Internal Medicine, Seoul National University Bundang Hospital, Seongnam-Si, Gyeonggi-Do Republic of Korea; 7 Division of Pulmonary and Critical Care Medicine, Department of Internal Medicine, Seoul Metropolitan Government-Seoul National University Boramae Medical Center, Seoul, Republic of Korea

**Keywords:** Pulmonary sclerosing pneumocytoma, Mortality, Surgery, Metastasis, Recurrence

## Abstract

**Background:**

Surgical resection is usually recommended for the treatment of pulmonary sclerosing pneumocytoma (PSP). However, no comparative study has demonstrated that surgical resection leads to improved outcomes. We aimed to compare all-cause mortality between patients with PSP who underwent surgery or did not and those without PSP.

**Methods:**

Participants aged ≥18 years who had pathologically diagnosed PSP between 2001 to 2018, at 3 hospitals were included. Randomly selected (up to 1:5) age-, sex-, and smoking status-matched controls without PSP who were randomly selected from those who underwent health checkups including chest CT were included. Mortality was compared using Kaplan–Meier estimates and Cox proportional hazards regression models. Literature review of studies reporting PSP was also conducted.

**Results:**

This study included 107 patients with PSP (surgery:non-surgery, 80:27) and 520 matched controls. There were no cases of lymph node or distant metastasis, recurrence, or mortality from PSP. No significant difference in all-cause mortality risk was observed between the PSP surgery, PSP non-surgery, and non-PSP groups (log rank test *P* = 0.78) (PSP surgery vs. non-PSP: adjusted hazards ratio [aHR], 1.80; 95% confidence interval [CI], 0.22–14.6; PSP non-surgery vs. non-PSP: aHR, 0.77; 95% CI, 0.15–3.86; PSP surgery vs. PSP non-surgery: aHR, 2.35; 95% CI, 0.20–28.2). In the literature review, we identified 3469 patients with PSP from 355 studies. Only 1.33% of these patients reported metastasis, recurrence, or death.

**Conclusions:**

All-cause mortality did not differ between patients with PSP and those without, irrespective of undergoing surgery. Our study and the literature review suggest that PSP has less impact on increased mortality risk.

**Supplementary Information:**

The online version contains supplementary material available at 10.1186/s12890-022-02199-1.

## Background

Pulmonary sclerosing pneumocytoma (PSP) is a rare neoplasm that was first described by Liebow and Hubbell in 1956 as sclerosing hemangioma [1]. Sclerosing hemangioma was renamed PSP and categorized into “adenoma” by World Health Organization in 2015 [2]. Although PSP was previously considered a vascular neoplasm, it is currently understood as a tumor originating from the primitive respiratory epithelium [3]. PSP is predominant in middle-aged women with increased prevalence in Asians [4, 5]. PSP is mainly found incidentally. More than 70% of patients with PSP are asymptomatic. Symptoms of PSP include hemoptysis, chronic cough, and chest pain [6]. It is generally observed as a slow-growing well-circumscribed nodule with high-strength homogeneous enhancement on computed tomography (CT) [7].

Surgical resection is usually recommended for the treatment of PSP [6] considering that cases with lymph node metastasis [3, 8–14], distant or pleural metastasis [15–17], recurrence [18], malignant transformation [19] and death [20] have been reported. However, as most patients with PSP show a benign indolent course, not all patients with PSP undergo surgical resection clinically [21]. In fact, it has not been clarified whether surgical resection leads to better outcomes. No study has compared outcomes between patients with PSP who underwent surgery and those who did not. In addition, it has not been investigated whether those with PSP have a higher mortality risk than those without PSP. Therefore, we aimed to investigate the treatment outcomes and prognosis of patients with PSP, including those who underwent surgery and those who did not.

## Methods

### Study design and participants

Participants who were diagnosed with PSP pathologically confirmed at tertiary hospitals (Seoul National University Hospital, Seoul National University Bundang Hospital and Seoul National University Seoul Metropolitan Government Boramae Medical Center) between January 1, 2001, and December 31, 2018 (“PSP group”) were enrolled. Those who were under 18 years of age and who lacked mortality information were excluded. Participants in the PSP group were categorized into “PSP surgery group” and “PSP non-surgery group” based on the treatment undergone for PSP. Participants in the PSP group were matched with up to five age-, sex-, and smoking status-matched controls without PSP who were randomly selected from those who underwent health checkups including chest CT at a health screening center (Seoul National University Healthcare System Gangnam Center) between October 2004 and December 2013 (“non-PSP group”). In the control group, those who presented with nodules suspicious of PSP on chest CT and those who had undergone lung surgery were excluded.

Their medical records, operative procedures, histological examinations, and outpatient clinic follow-up data were reviewed retrospectively. In the PSP group, we investigated age, sex, body mass index (BMI), smoking status, comorbidity, first department visit, initial symptoms, and tumor size, location, and number. In addition, we analyzed initial diagnostic methods, surgery methods, whether the lymph node was enlarged on chest CT, and whether lymph nodes were dissected during surgery, as well as the presence of lymph node metastasis, distant metastasis, and recurrence. In the non-PSP group, we collected data on age, sex, BMI, smoking status, and comorbidity. The index date for statistics was defined as the date of the first biopsy or surgery performed in the PSP group and chest CT performed in the non-PSP group. Comorbidities were identified based on the International Classification of Diseases, 10th Revision codes registered before the index date. Comorbidities were classified as follows: hypertension (I10), diabetes mellitus (E10–E14), dyslipidemia (E78), respiratory diseases, cardiovascular diseases (I20–I25, I50, I110), chronic liver diseases (K703, except K7039, K717, K740–746, K761, P788), chronic kidney diseases (E10.2x, E11.2x, E13.2x, E14.2x, I12.0, I12.9, I13.x, N18, N19), and malignancy (C00–C97). Respiratory diseases were investigated in patients with pulmonary tuberculosis (A15, A16, A19, B909), nontuberculous mycobacterial pulmonary disease (A31, except A311), chronic obstructive pulmonary disease (J44), asthma (J45 and J46), bronchiectasis (J47, Q334), and interstitial lung disease (J70, J84).

The primary outcome was all-cause mortality. Mortality information, including that on the causes of death, was collected from the Korean National Statistical Office. This study was approved and the requirement for written informed consent was waived by the institutional review board of Seoul National University Hospital (IRB no. H-1607-038-774). The study was conducted in accordance with the tenets of the Declaration of Helsinki.

### Statistical analysis

We compared the baseline characteristics between the PSP surgery group and PSP non-surgery group*s* using Student’s *t*-tests for continuous variables and chi-squared tests or Fisher’s exact tests for categorical variables. Paired t-tests and McNemar tests were used to compare the PSP group and the matched non-PSP group. Kaplan–Meier curves with log-rank tests and Cox proportional hazards regression adjusted by covariates were used to compare the all-cause mortality risk between the groups. A proportional hazards assumption was also tested using Schoenfeld residuals. The estimates of mortality risk were presented as adjusted hazard ratios (aHRs) and their 95% confidence intervals (CIs). Statistical significance was set at *p* <  0.05. All analyses were performed using Stata 14.2 (StataCorp., College Station, TX, USA) and SPSS version 26 (IBM, Armonk, NY, USA).

### Literature review

We conducted a literature review of studies that reported patients with PSP. We identified studies on PSP using the terms “pulmonary sclerosing pneumocytoma,” “pulmonary sclerosing hemangioma,” “pneumocytoma of the lung,” and “sclerosing hemangioma of the lung” on PUBMED. Original articles, case reports, case series, and review articles that reported the total number of patients and the number of metastases, recurrence, or death were included in the review. After removing duplicate data, we counted the number of patients who reported with PSP, lymph node metastases, distant metastases, or recurrence and the global mortality rate of PSP since the first PSP case was reported in 1956 [1].

## Results

### Participants

**Fig. 1:**
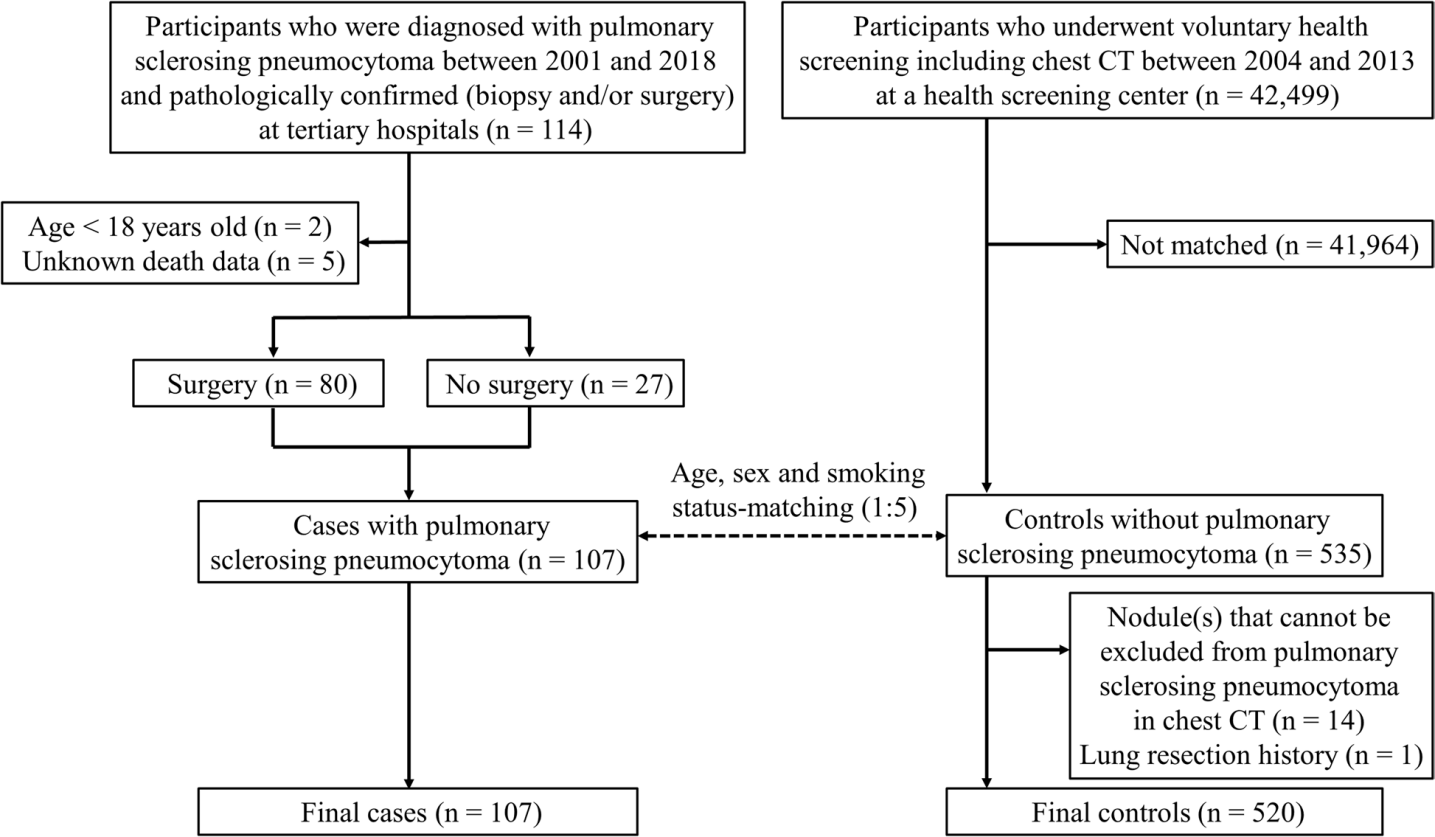
Study flow diagram

The study flow diagram is shown in Fig. 1. A total of 107 patients with PSP were included. Most (91.6%) were women, and the mean age was 50.6 ± 12.5 years. Of these, 80 patients underwent surgery (PSP surgery group) and 27 patients were categorized into the PSP non-surgery group, and there were no significant differences in baseline characteristics (Table 1).

**Table 1:** Baseline characteristics of patients with pulmonary sclerosing pneumocytoma

Baseline characteristics	PSP surgery group (***N*** = 80)	PSP non-surgery group (***N*** = 27)	Total (***N*** = 107)	***P***
**Age, years**	50.9 ± 12.7	49.8 ± 11.9	50.6 ± 12.5	0.684
**Female**	72 (90.0)	26 (96.3)	98 (91.6)	0.444
**Body mass index, kg/m**^**2**^	22.5 ± 3.2	24.0 ± 3.4	23.0 ± 3.2	0.358
**Never Smoker**	71 (88.8)	22 (81.5)	93 (86.9)	0.337
**Comorbidities**				
**Hypertension**	1 (1.2)	1 (3.7)	2 (1.9)	0.443
**Diabetes**	0 (0.0)	1 (3.7)	1 (0.9)	0.252
**Dyslipidemia**	2 (2.5)	0 (0.0)	2 (1.9)	1.000
**Respiratory diseases**	1 (1.2)	0 (0.0)	1 (0.9)	1.000
**Cardiovascular diseases**	5 (6.2)	0 (0.0)	5 (4.7)	0.327
**Chronic liver diseases**	1 (1.2)	0 (0.0)	1 (0.9)	1.000
**Chronic kidney diseases**	0 (0.0)	0 (0.0)	0 (0.0)	1.000
**Malignancy**	21 (26.2)	8 (29.6)	29 (27.1)	0.927

Continuous variables are expressed as mean ± standard deviation

Categorical variables are expressed as number (%)

A total of 520 age-, sex-, and smoking status-matched controls were included. The baseline characteristics of the PSP and non-PSP groups are summarized in Supplementary Table S1. The PSP group had a higher mean BMI and higher rates of diabetes mellitus, cardiovascular diseases, chronic liver diseases, and malignancy.

### Tumor characteristics of PSP group

The tumor characteristics of patients in the PSP group are summarized in Table 2. The rate of not performing surgery was significantly higher when the patient first visited the pulmonology department than the thoracic surgery department. The majority (79.4%) of the patients had no symptoms and the remaining 20.6% had symptoms. Cough was the most common symptom at 9.3%, and hemoptysis occurred in 2.8%. Three out of 107 patients (2.8%) underwent surgery due to repeated symptoms that reduced their quality of life. All symptoms were hemoptysis, and the hemoptysis improved after surgery in all three patients. In particular, there was one person who had a blood-tinged sputum that repeats for about a month every year. Eventually, the operation was performed and the blood-tinged sputum improved. Less than a fifth had a tumor size > 3 cm. There were no significant differences in the presence of symptoms and tumor size between the PSP surgery group and PSP non-surgery group*s*. Most of them were single lesions, but four patients (3.7%) showed two lesions, and all of them underwent surgery. In the PSP surgery group, one-third of the patients were diagnosed by either percutaneous needle biopsy (*n* = 24) or bronchoscopic biopsy (*n* = 3), including two cases confirmed by endobronchial ultrasound-guided transbronchial needle aspiration (EBUS-TBNA), before surgery. Lymph node enlargement was observed on chest CT in seven patients. In the PSP surgery group, lymph node dissection was performed in 47.5% of the patients. During diagnosis and follow-up over 87.4 ± 52.0 months, there were no cases of lymph node metastasis, distant metastasis, or recurrence.

**Table 2:** Tumor characteristics of patients with pulmonary sclerosing pneumocytoma

Characteristics	PSP surgery group (***N*** = 80)	PSP non-surgery group (***N*** = 27)	Total (***N*** = 107)	***P***
**Initial department**				0.001
**Pulmonology**	43 (53.8)	25 (92.6)	68 (63.6)	
**Thoracic Surgery**	37 (46.2)	2 (7.4)	39 (36.4)	
**Symptom**				0.617
**No symptom**	60 (75.0)	25 (92.6)	85 (79.4)	
**Cough**	9 (11.2)	1 (3.7)	10 (9.3)	
**Hemoptysis**	3 (3.8)	0 (0.0)	3 (2.8)	
**Chest discomfort/pain**	5 (6.2)	1 (3.7)	6 (5.6)	
**Sputum**	5 (6.2)	0 (0.0)	5 (4.7)	
**Dyspnea**	4 (5.0)	0 (0.0)	4 (3.7)	
**Fever**	1 (1.2)	0 (0.0)	1 (0.9)	
**Tumor size (cm)**	2.0 (1.3–2.0)	1.7 (1.5–2.3)	1.9 (1.5–2.5)	0.541
**Tumor size**				0.334
**< 1 cm**	6 (7.5)	0 (0.0)	6 (5.6)	
**1–2 cm**	37 (46.2)	16 (59.3)	53 (49.5)	
**2–3 cm**	24 (30.0)	9 (33.3)	33 (30.8)	
**3–4 cm**	12 (15.0)	1 (3.7)	13 (12.1)	
**4–5 cm**	3 (3.8)	0 (0.0)	3 (2.8)	
**≥ 5 cm**	2 (2.5)	1 (3.7)	3 (2.8)	
**Tumor location**				0.405
**Right upper lobe**	6 (7.5)	4 (14.8)	10 (9.3)	
**Right middle lobe**	19 (23.8)	8 (29.6)	27 (25.2)	
**Right lower lobe**	15 (18.8)	7 (25.9)	22 (20.6)	
**Left upper lobe**	15 (18.8)	3 (11.1)	18 (16.8)	
**Left lower lobe**	27 (33.8)	5 (18.5)	32 (29.9)	
**Tumor Number**				0.570
**Single**	76 (95.0)	27 (100.0)	103 (96.3)	
**Two**	4 (5.0)	0 (0.0)	4 (3.7)	
**Initial diagnostic methods**				< 0.001
**Percutaneous needle biopsy**	24 (30.0)	27 (100.0)	51 (47.7)	
**Surgery**	53 (66.2)	0 (0.0)	53 (49.5)	
**Bronchoscopic biopsy**^a^	3 (3.7)	0 (0.0)	3 (2.8)	
**Surgery methods (only for surgery group)**				
**Pneumonectomy**	0 (0.0)			
**Lobectomy**	24 (30.0)			
**Segmentectomy**	12 (15.0)			
**Wedge resection**	44 (55.0)			
**Enucleation**	1 (1.2)			
**Lymph node enlargement on CT**	6 (7.5)	1 (3.7)	7 (6.5)	0.676
**Lymph node dissection during surgery** **(only for surgery group)**	38 (47.5)			
**Lymph node metastasis (only for surgery group)**	0 (0.0)			
**Distant metastasis**	0 (0.0)	0 (0.0)	0 (0.0)	
**Recurrence**	0 (0.0)	0 (0.0)	0 (0.0)	

Continuous variables are expressed as median (interquartile range)

Categorical variables are expressed as number (%)

*CT* Computed tomography

^a^ Two of them were diagnosed with endobronchial ultrasound guided-transbronchial needle aspiration for lung nodules, not lymph nodes

### All-cause mortality

**Fig. 2:**
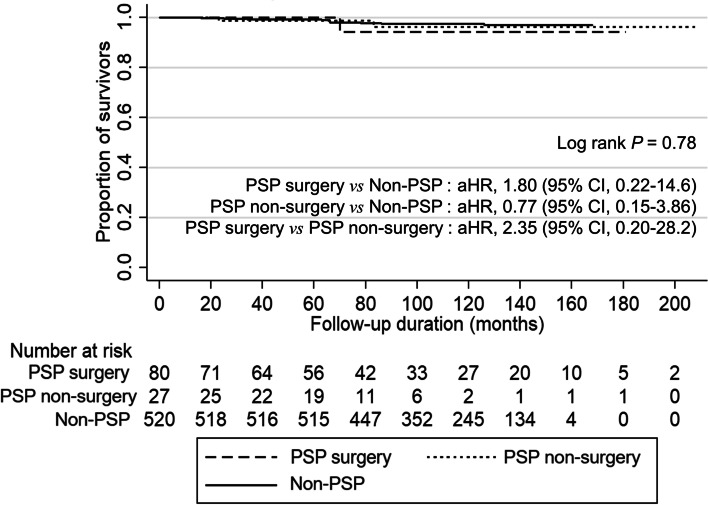
Comparisons of all-cause mortality risk between groups. PSP, pulmonary sclerosing pneumocytoma

Two (1.86%) deaths in the PSP surgery group (91.5 ± 55.5 months) and one (0.93%) death in the PSP non-surgery group (75.1 ± 38.1 months) were observed during a follow-up period of 87.4 ± 52.0 months. There were no cases of PSP-related deaths. There were 14 deaths in the non-PSP group. There was no significant difference in all-cause mortality risk between the PSP surgery group, PSP non-surgery group, and non-PSP group based on Kaplan–Meier estimates (log-rank test, *P* = 0.78). Multivariable Cox proportional hazards regression also showed that there were no differences in all-cause mortality risk between groups, even after adjustment for age, sex, BMI, smoking status, and comorbidities (PSP surgery group vs. non-PSP group: aHR, 1.80; 95% CI, 0.22–14.6; PSP non-surgery group vs. non-PSP group: aHR, 0.77; 95% CI, 0.15–3.86; PSP surgery group vs. PSP non-surgery group: aHR, 2.35; 95% CI, 0.20–28.2). An assumption of proportional hazards was not rejected (*P* > 0.99) (Fig. 2).

### Literature review

We reviewed all PSP cases, lymph node metastases, distant metastases, recurrence, and deaths published worldwide over 65 years since the first case of PSP reported in 1956 (Supplementary Table S2). We identified 355 studies that reported the total number of patients and the number of metastases, recurrence, or death. To date, 3469 patients with PSP have been reported. Among them, there were 38 cases with lymph node metastases, 6 distant and pleural metastases, 4 recurrences, and 1 death.

## Discussion

The principal finding of the current study was that there were no differences in all-cause mortality risk between the PSP surgery group, PSP non-surgery group, and non-PSP group. To our knowledge, this is the first study to investigate the all-cause mortality risk of patients with PSP based on the treatment undergone compared with those without PSP. To date, the all-cause mortality risk of patients with PSP has not been reported, although mortality is the most important factor in treatment outcomes and prognosis in patients with tumors. In addition, no studies have compared outcomes based on undergoing surgical resection by the patients with PSP. Although surgical resection has been considered a treatment option [6, 14], the results of the present study questions the validity of surgical resection in all cases of PSP. The PSP surgery group did not show better survival than the PSP non-surgery group in our study. The two groups had similar baseline characteristics, including age, sex, smoking status, comorbidities, tumor size, and lymph node enlargement. Even the PSP non-surgery group showed a comparable all-cause mortality risk to age-, sex-, and smoking status-matched non-PSP control group.

The small impact of PSP on all-cause mortality might be explained by the benign nature of the tumor. There were no PSP-related deaths among the participants of our study. But one case of PSP-related death was identified through an extensive literature review [20]. It was the first case of death from respiratory and circulatory failure due to a large, multiple PSP tumor and lymph node and extrapulmonary metastases (liver, abdominal cavity, bone) compressing mediastinal tissue. In addition, we could identify only one serious PSP patient from the literature reviews, who had experienced respiratory arrest due to airway obstruction by endobronchial PSP, which improved after pneumonectomy [22].

The main reason for surgical resection of PSP is the suspicion of early-stage lung cancer. In 1986, Tanaka et al. reported the first case of PSP with lymph node metastasis [8]. In a relatively large series of published PSP cases, only 1 out of 100 and 3 out of 239 PSP patients showed lymph node metastases [3, 13]. These reports suggested that PSP may be potentially malignant. When we extensively searched all references regarding PSP, we identified a total of 38 cases of PSP with lymph node metastases [3, 8–14], 6 with distant and pleural metastases [15–17], 4 with recurrence [18], 1 with massive necrosis and vascular invasion [23] and 1 with death [20]. However, considering that 3469 PSP cases were reported in the past 65 years after the reporting of the first PSP case, only 1.33% of PSP cases can be regarded as malignant. Interestingly, there was only one death due to PSP in these patients presenting with metastasis or recurrence, suggesting that PSP does not have a significant impact on prognosis even if metastasis or recurrence occurs. Recently, a case of malignant transformation in both cuboidal surface cells and stromal round cells was confirmed for the first time, but it was reported as an outpatient follow-up state without recurrence or metastasis after surgery [19]. Rather, lung resection surgery might lead to significant complications, including prolonged air leak, bronchopleural fistula, pneumonia, acute respiratory failure, hemorrhage, atelectasis, pneumothorax, bronchospasm, pulmonary embolism, acute respiratory distress syndrome, and cardiovascular complications [24] affecting prognosis, although there were no deaths immediately after surgery in our study. Therefore, it could be helpful to identify the risk group requiring surgery among patients with PSP. Among the prior studies related to this, it was reported that PSP patients with spindle cells or male patients may be more prone to metastasis [13]. Another study reported that young male patients are prone to lymph node metastasis, and the tumor size is larger [25]. Moreover, resection has been performed to confirm the diagnosis in lesions that were otherwise not possible by endoscopic or percutaneous biopsy [26], to relieve local compression of adjacent structures [27, 28], or in the setting of mixed histology of PSP combined with another more aggressive tumor [29]. However, in the present study, we could not determine surgery candidates because there was no patient who presented with metastasis, recurrence, or death from PSP. Further studies related to the surgical indications are required.

The strengths of the current study are as follows. This is the first study to investigate the all-cause mortality risk of patients with PSP. A long-term follow-up duration (87.4 ± 52.0 months) is another advantage of our study. Comorbidities were included as confounders of mortality risk in the multivariate analysis. We also reviewed reports on PSP cases, lymph nodes, distant metastases, recurrence, and deaths published worldwide in 65 years since the first PSP case was reported.

## Conclusions

In conclusion, PSP did not affect all-cause mortality, regardless of whether surgery was performed, compared to those without PSP. Our study and the literature review suggest that PSP has less impact on an increased mortality risk. Efforts should be made through further studies to identify subgroups that actually require surgery rather than performing surgical resection in all patients with PSP.

## Supplementary Information

**Additional file 1: Table S1.** Characteristics of patients with pulmonary sclerosing pneumocytoma and age-, sex-, and smoking status-matched controls. **Table S2.** Review of pulmonary sclerosing pneumocytoma cases with lymph node metastases, distant metastases, recurrence, and deaths published worldwide.

## Data Availability

The datasets used and/or analysed during the current study are available from the corresponding author on reasonable request.

## Abbreviations

PSP: Pulmonary sclerosing pneumocytoma
CT: Computed tomography
BMI: Body mass index
