# Supplementary material for: Pulmonary sclerosing pneumocytoma and mortality risk
Source: BMC Pulm Med. 2022 Nov 7;22:404. doi: 10.1186/s12890-022-02199-1 (PMC9641959; doi:10.1186/s12890-022-02199-1)
Supplement: Supplementary file 1 — Additional file 1: Table S1. Characteristics of patients with pulmonary sclerosing pneumocytoma and age-, sex-, and smoking status-matched controls. Table S2. Review of pulmonary sclerosing pneumocytoma cases with lymph node metastases, distant metastases, recurrence, and deaths published worldwide. [file 12890_2022_2199_MOESM1_ESM.docx]

**Table S1**. Characteristics of patients with pulmonary sclerosing pneumocytoma and age-, sex-, and smoking status-matched controls.

| **Baseline characteristics** | **PSP group**  **(N = 107)** | **Non-PSP group**  **(N = 520)** | **Total**  **(N = 627)** | ***P*** |
| --- | --- | --- | --- | --- |
| **Age, years** | 51.0 ± 12.5 | 50.4 ± 12.4 | 50.5 ± 12.4 |  |
| **Female** | 98 (91.6) | 475 (91.3) | 573(91.4) |  |
| **Body mass index, kg/m^2^** | 23.0 ± 3.2 | 22.0 ± 3.8 | 22.0 ± 3.7 | < 0.001 |
| **Never smoker** | 14 (13.1) | 70 (13.5) | 84 (13.4) |  |
| **Comorbidities** |  | | |  |
| **Hypertension** | 2 (1.9) | 12 (2.3) | 14 (2.2) | 0.670 |
| **Diabetes** | 1 (0.9) | 1 (0.2) | 2 (0.3) | 0.007 |
| **Dyslipidemia** | 2 (1.9) | 11 (2.1) | 13 (2.1) | 0.549 |
| **Respiratory diseases** | 1 (0.9) | 12 (2.3) | 13 (2.1) | 0.090 |
| **Cardiovascular diseases** | 5 (4.7) | 5 (1.0) | 10 (1.6) | < 0.001 |
| **Chronic liver** **diseases** | 1 (0.9) | 0 (0.0) | 1 (0.2) | 0.025 |
| **Chronic kidney diseases** | 0 (0.0) | 0 (0.0) | 0 (0.0) |  |
| **Malignancy** | 29 (27.1) | 20 (3.8) | 49 (7.8) | < 0.001 |

Continuous variables are expressed as mean ± standard deviation.

Categorical variables are expressed as number (%).

PSP, pulmonary sclerosing pneumocytoma

**Table S2**. Review of pulmonary sclerosing pneumocytoma cases with lymph node metastases, distant metastases, recurrence, and deaths published worldwide

|  | **PMID** | **First author** | **Published year** | **Country** | **Total patients** | **LN metastasis** | **Distant /pleural metastasis** | **Recurrence** | **Death** | **Language**^1^ |
| --- | --- | --- | --- | --- | --- | --- | --- | --- | --- | --- |
| **Total** |  |  |  |  | **N =3,469** | **n = 38** | **n = 6** | **n = 4** | **n = 1** |  |
| 1 | 32022435 | Zahra Maleki | 2020 | United States | 9 | 0 | 0 | 0 | 0 |  |
| 2 | 30247298 | [Estefania Rivera](https://pubmed.ncbi.nlm.nih.gov/?term=Rivera+E&cauthor_id=30247298) | 2018 | United States | 1 | 0 | 0 | 0 | 0 |  |
| **3** | **30309107** | [Jason Han](https://pubmed.ncbi.nlm.nih.gov/?term=Han+J&cauthor_id=30309107) | 2018 | Australia | 1 | 0 | 0 | 0 | 0 |  |
| 4 | **25634202** | [So Youn Shin](https://pubmed.ncbi.nlm.nih.gov/?term=Shin+SY&cauthor_id=25634202) | 2015 | South Korea | 76 | 1 | 0 | 1^2^ | 0 |  |
| 5 | 32590790 | [Huu Y Le](https://pubmed.ncbi.nlm.nih.gov/?term=Le+HY&cauthor_id=32590790) | 2020 | Vietnam | 1 | 0 | 0 | 0 | 0 |  |
| 6 | 31194169 | [Ko Lee](https://pubmed.ncbi.nlm.nih.gov/?term=Lee+K&cauthor_id=31194169) | 2019 | Japan | 1 | 0 | 0 | 0 | 0 |  |
| 7 | 32397805 | [Livio Solari](https://pubmed.ncbi.nlm.nih.gov/?term=Solari+L&cauthor_id=32397805) | 2020 | Belgium | 1 | 0 | 0 | 0 | 0 |  |
| 8 | 30847275 | [Burcu Yalcin](https://pubmed.ncbi.nlm.nih.gov/?term=Yalcin+B&cauthor_id=30847275) | 2019 | Turkey | 1 | 0 | 0 | 0 | 0 |  |
| 9 | 32666331 | [Gökhan Kocaman](https://pubmed.ncbi.nlm.nih.gov/?term=Kocaman+G&cauthor_id=32666331) | 2021 | Turkey | 5 | 1 | 0 | 0 | 0 |  |
| 10 | [30891111](https://www.ncbi.nlm.nih.gov/pubmed/30891111) | [Kazuhiko Morikawa](https://www.ncbi.nlm.nih.gov/pubmed/?term=Morikawa%20K%5BAuthor%5D&cauthor=true&cauthor_uid=30891111) | 2019 | Japan | 1 | 0 | 0 | 0 | 0 |  |
| 11 | 30691016 | [Aleksandra Lovrenski](https://pubmed.ncbi.nlm.nih.gov/?term=Lovrenski+A&cauthor_id=30691016) | 2019 | Serbia | 6 | 0 | 0 | 0 | 0 |  |
| 12 | [31428771](https://www.ncbi.nlm.nih.gov/pubmed/31428771) | [Takashi Sakai](https://www.ncbi.nlm.nih.gov/pubmed/?term=Sakai%20T%5BAuthor%5D&cauthor=true&cauthor_uid=31428771), | 2019 | Japan | 1 | 0 | 0 | 0 | 0 |  |
| 13 | 27761142 | [Jui-Hung Hung](https://pubmed.ncbi.nlm.nih.gov/?term=Hung+JH&cauthor_id=27761142) | 2016 | Taiwan | 1 | 0 | 0 | 0 | 0 |  |
| 14 | 28881050 | [Cheng-Han Yang](https://pubmed.ncbi.nlm.nih.gov/?term=Yang+CH&cauthor_id=28881050) | 2018 | Taiwan | 59 | 0 | 0 | 0 | 0 |  |
| 15 | 33369106 | [Xiao-Qiong Ni](https://pubmed.ncbi.nlm.nih.gov/?term=Ni+XQ&cauthor_id=33369106) | 2021 | China | 25 | 0 | 0 | 0 | 0 |  |
| 16 | [30964602](https://www.ncbi.nlm.nih.gov/pubmed/30964602) | [Kyungsoo Bae](https://www.ncbi.nlm.nih.gov/pubmed/?term=Bae%20K%5BAuthor%5D&cauthor=true&cauthor_uid=30964602), | 2019 | South Korea | 1 | 0 | 0 | 0 | 0 |  |
| 17 | 31131992 | [Jie Xu](https://pubmed.ncbi.nlm.nih.gov/?term=Xu+J&cauthor_id=31131992) | 2019 | China, | 136 | 0 | 0 | 0 | 0 |  |
| 18 | 30985653 | [Akriti Khanna](https://pubmed.ncbi.nlm.nih.gov/?term=Khanna+A&cauthor_id=30985653) | 2019 | United States | 1 | 0 | 0 | 0 | 0 |  |
| 19 | 32868166 | [Y Hassani](https://pubmed.ncbi.nlm.nih.gov/?term=Hassani+Y&cauthor_id=32868166) | 2020 | France | 2 | 0 | 0 | 0 | 0 | French |
| 20 | 28825313 | [Ing Xiang Soo](https://pubmed.ncbi.nlm.nih.gov/?term=Soo+IX&cauthor_id=28825313) | 2017 | Singapore | 1 | 1 | 0 | 0 | 0 |  |
| 21 | 31861007 | [Xu LuLu](https://pubmed.ncbi.nlm.nih.gov/?term=LuLu+X&cauthor_id=31861007) | 2019 | China | 1 | 0 | 0 | 0 | 0 |  |
| 22 | 33155300 | [Konstantinos Kosmas](https://pubmed.ncbi.nlm.nih.gov/?term=Kosmas+K&cauthor_id=33155300) | 2021 | Greece | 1 | 0 | 0 | 0 | 0 |  |
| 23 | 30702609 | [Zhao Wang](https://pubmed.ncbi.nlm.nih.gov/?term=Wang+Z&cauthor_id=30702609) | 2019 | China, | 1 | 0 | 0 | 0 | 0 |  |
| 24 | 33357311 | [Xiaojing Liu](https://pubmed.ncbi.nlm.nih.gov/?term=Liu+X&cauthor_id=33357311) | 2020 | China, | 35 | 0 | 0 | 0 | 0 | Chinese |
| 25 | 27302690 | [Russell Wb Fayers](https://pubmed.ncbi.nlm.nih.gov/?term=Fayers+RW&cauthor_id=27302690) | 2016 | Australia | 1 | 0 | 0 | 0 | 0 |  |
| 26 | 32441345 | [Qiqi Gao](https://pubmed.ncbi.nlm.nih.gov/?term=Gao+Q&cauthor_id=32441345) | 2020 | China | 239 | 3 | 0 | 0 | 0 |  |
| 27 | 29524065 | [Yuki Shiina](https://pubmed.ncbi.nlm.nih.gov/?term=Shiina+Y&cauthor_id=29524065) | 2018 | Japan | 1 | 0 | 0 | 0 | 0 |  |
| 28 | 27385997 | [Jung-Hwan Lim](https://pubmed.ncbi.nlm.nih.gov/?term=Lim+JH&cauthor_id=27385997) | 2016 | South Korea | 1 | 0 | 0 | 0 | 0 |  |
| 29 | 31527710 | [Yi-Chen Yeh](https://pubmed.ncbi.nlm.nih.gov/?term=Yeh+YC&cauthor_id=31527710) | 2020 | Taiwan | 44 | 0 | 0 | 0 | 0 |  |
| 30 | 28398699 | [Erika Hissong](https://pubmed.ncbi.nlm.nih.gov/?term=Hissong+E&cauthor_id=28398699) | 2017 | United States | 1 | 0 | 0 | 0 | 0 |  |
| 31 | 28249927 | [Jain Zhou](https://pubmed.ncbi.nlm.nih.gov/?term=Zhou+J&cauthor_id=28249927) | 2017 | United States | 1 | 0 | 0 | 0 | 0 |  |
| 32 | 31166879 | [Youssef Nasr](https://pubmed.ncbi.nlm.nih.gov/?term=Nasr+Y&cauthor_id=31166879) | 2019 | Canada | 1 | 0 | 0 | 0 | 0 |  |
| 33 | 29221270 | [Jun Zhu](https://pubmed.ncbi.nlm.nih.gov/?term=Zhu+J&cauthor_id=29221270) | 2017 | China | 187 | 0 | 0 | 0 | 0 |  |
| 34 | 27761142 | [Jui-Hung Hung](https://pubmed.ncbi.nlm.nih.gov/?term=Hung+JH&cauthor_id=27761142) | 2016 | Taiwan | 1 | 0 | 0 | 0 | 0 |  |
| 35 | 24667045 | [So Youn Shin](https://pubmed.ncbi.nlm.nih.gov/?term=Shin+SY&cauthor_id=24667045) [^1^](https://pubmed.ncbi.nlm.nih.gov/24667045/#affiliation-1) | 2014 | South Korea | 1 | 0 | 0 | 0 | 0 |  |
| 36 | 30147108 | [Tong-Tong Li](https://pubmed.ncbi.nlm.nih.gov/?term=Li+TT&cauthor_id=30147108) | 2018 | China | 1 | 0 | 0 | 0 | 0 |  |
| 37 | 28474862 | [Hwa Jin Cho](https://www.ncbi.nlm.nih.gov/pubmed/?term=Cho%20HJ%5BAuthor%5D&cauthor=true&cauthor_uid=28474862), | 2017 | South Korea | 1 | 0 | 0 | 0 | 0 |  |
| 38 | 29460895 | [IV Dvorakovskaya](https://pubmed.ncbi.nlm.nih.gov/?term=Dvorakovskaya+IV&cauthor_id=29460895) | 2018 | Russia | 6 | 0 | 0 | 0 | 0 | Russian |
| 39 | 32913868 | [Xiao-Yu Han](https://pubmed.ncbi.nlm.nih.gov/?term=Han+XY&cauthor_id=32913868) | 2020 | China | 1 | 0 | 0 | 0 | 0 |  |
| 40 | 32984377 | [Beatrice Aramini](https://pubmed.ncbi.nlm.nih.gov/?term=Aramini+B&cauthor_id=32984377) | 2020 | Italy | 5 | 1 | 0 | 1^3^ | 0 |  |
| 41 | 21471259 | [Su-Ying Low](https://pubmed.ncbi.nlm.nih.gov/?term=Low+SY&cauthor_id=21471259) | 2011 | Singapore | 19 | 1 | 0 | 0 | 0 |  |
| 42 | **32317291** | [Prodipto Pal](https://pubmed.ncbi.nlm.nih.gov/?term=Pal+P&cauthor_id=32317291) | 2020 | Canada | 2 | 0 | 0 | 0 | 0 |  |
| 43 | 29930720 | [Lei Jiang](https://pubmed.ncbi.nlm.nih.gov/?term=Jiang+L&cauthor_id=29930720) | 2018 | China | 14 | 0 | 0 | 0 | 0 |  |
| 44 | 29696743 | [Yuko Kitawaki](https://pubmed.ncbi.nlm.nih.gov/?term=Kitawaki+Y&cauthor_id=29696743) | 2018 | Japan | 1 | 0 | 0 | 0 | 0 |  |
| 45 | 27346414 | [Saraswati Pokharel](https://pubmed.ncbi.nlm.nih.gov/?term=Pokharel+S&cauthor_id=27346414) | 2016 | United States | 1 | 1 | 0 | 0 | 0 |  |
| 46 | 23865575 | [Timothy Craig Allen](https://pubmed.ncbi.nlm.nih.gov/?term=Allen+TC&cauthor_id=23865575) | 2013 | United States | 1 | 0 | 0 | 0 | 0 |  |
| 47 | 31133161 | [Mustafa Kupeli](https://pubmed.ncbi.nlm.nih.gov/?term=Kupeli+M&cauthor_id=31133161) | 2019 | Turkey | 1 | 0 | 0 | 0 | 0 |  |
| 48 | 15731902 | [B M Einsfelder](https://pubmed.ncbi.nlm.nih.gov/?term=Einsfelder+BM&cauthor_id=15731902) | 2005 | Germany | 8 | 0 | 0 | 0 | 0 | German |
| - 49 | 18088044 | [Jelena Stojsić](https://pubmed.ncbi.nlm.nih.gov/?term=Stojsi%C4%87+J&cauthor_id=18088044) | 2007 | Serbia | 1 | 0 | 0 | 0 | 0 | Serbian |
| - 50 | 30971157 | [Zhu-Qing Yuan](https://pubmed.ncbi.nlm.nih.gov/?term=Yuan+ZQ&cauthor_id=30971157) | 2019 | China | 1 | 0 | 0 | 0 | 0 |  |
| 51 | 30004067 | [Xiaojun Wang](https://pubmed.ncbi.nlm.nih.gov/?term=Wang+X&cauthor_id=30004067) | 2018 | China | 1 | 1 | 0 | 0 | 0 |  |
| - 52 | 12653576 | [Aya Miyagawa-Hayashino](https://pubmed.ncbi.nlm.nih.gov/?term=Miyagawa-Hayashino+A&cauthor_id=12653576) | 2003 | Japan | 4 | 4 | 0 | 0 | 0 |  |
| 53 | 16902885 | [T Komatsu](https://pubmed.ncbi.nlm.nih.gov/?term=Komatsu+T&cauthor_id=16902885) | 2006 | Japan | 1 | 0 | 0 | 0 | 0 |  |
| 54 | 15561002 | [Akira Iyoda](https://pubmed.ncbi.nlm.nih.gov/?term=Iyoda+A&cauthor_id=15561002) | 2004 | Japan | 26 | 0 | 0 | 0 | 0 |  |
| 55 | 23587094 | [Bojiang Chen](https://pubmed.ncbi.nlm.nih.gov/?term=Chen+B&cauthor_id=23587094) | 2013 | China | 26 | 0 | 0 | 0 | 0 |  |
| 56 | 28384875 | [Vasugi Gramani Arumugam](https://pubmed.ncbi.nlm.nih.gov/?term=Arumugam+VG&cauthor_id=28384875) | 2017 | India | 1 | 0 | 0 | 0 | 0 |  |
| 57 | 24944657 | [Yasushi Adachi](https://pubmed.ncbi.nlm.nih.gov/?term=Adachi+Y&cauthor_id=24944657) | 2014 | Japan | 1 | 1 | 0 | 0 | 0 |  |
| 58 | 21264765 | [Hidemi Suzuki](https://pubmed.ncbi.nlm.nih.gov/?term=Suzuki+H&cauthor_id=21264765) | 2011 | Japan | 1 | 0 | 1^4^ | 0 | 0 |  |
| 59 | 27749415 | [Alex Cheen Hoe Khoo](https://pubmed.ncbi.nlm.nih.gov/?term=Khoo+AC&cauthor_id=27749415) | 2017 | Malaysia. | 1 | 0 | 0 | 0 | 0 |  |
| 60 | 27231179 | [Ai-Min Hu](https://pubmed.ncbi.nlm.nih.gov/?term=Hu+AM&cauthor_id=27231179) | 2016 | China | 46 | 0 | 0 | 0 | 0 |  |
| 61 | 31775674 | [Xiao Teng](https://pubmed.ncbi.nlm.nih.gov/?term=Teng+X&cauthor_id=31775674) | 2019 | China | 1 | 0 | 0 | 0 | 0 |  |
| 62 | 22800519 | [Yu Sun](https://pubmed.ncbi.nlm.nih.gov/?term=Sun+Y&cauthor_id=22800519) | 2012 | China | 49 | 0 | 0 | 0 | 0 | Chinese |
| 63 | 29451344 | [Hitomi Kawai](https://pubmed.ncbi.nlm.nih.gov/?term=Kawai+H&cauthor_id=29451344) | 2018 | Japan | 1 | 0 | 0 | 0 | 0 |  |
| 64 | 31966733 | [Lisha Jiang](https://pubmed.ncbi.nlm.nih.gov/?term=Jiang+L&cauthor_id=31966733) | 2017 | China | 1 | 0 | 0 | 0 | 0 |  |
| 65 | 26175598 | [Yong Pyo Kim](https://pubmed.ncbi.nlm.nih.gov/?term=Kim+YP&cauthor_id=26175598) | 2015 | South Korea | 1 | 0 | 0 | 0 | 0 |  |
| 66 | 29236566 | [Xiangshan Fan](https://pubmed.ncbi.nlm.nih.gov/?term=Fan+X&cauthor_id=29236566) | 2017 | China | 1 | 0 | 0 | 0 | 0 |  |
| 67 | 27902879 | [Naoto Kuroda](https://pubmed.ncbi.nlm.nih.gov/?term=Kuroda+N&cauthor_id=27902879) | 2017 | Japan | 3 | 0 | 0 | 0 | 0 |  |
| 68 | 31546071 | [Guanming Jiang](https://pubmed.ncbi.nlm.nih.gov/?term=Jiang+G&cauthor_id=31546071) | 2019 | China | 1 | 0 | 0 | 0 | 0 |  |
| 69 | 16677478 | [Bei-li Gao](https://pubmed.ncbi.nlm.nih.gov/?term=Gao+BL&cauthor_id=16677478) | 2006 | China | 15 | 0 | 0 | 0 | 0 | Chinese |
| 70 | 26552471 | [Min Kyoung Kim](https://pubmed.ncbi.nlm.nih.gov/?term=Kim+MK&cauthor_id=26552471) | 2015 | South Korea | 1 | 1 | 1^5^ | 0 | 0 |  |
| 71 | 28166143 | [Giordano Savelli](https://pubmed.ncbi.nlm.nih.gov/?term=Savelli+G&cauthor_id=28166143) | 2017 | Italy | 1 | 0 | 0 | 0 | 0 |  |
| 72 | 2548701 | [Y Satoh](https://pubmed.ncbi.nlm.nih.gov/?term=Satoh+Y&cauthor_id=2548701) | 1989 | Japan | 3 | 0 | 0 | 0 | 0 |  |
| 73 | 33165428 | [Li Cai](https://pubmed.ncbi.nlm.nih.gov/?term=Cai+L&cauthor_id=33165428) | 2020 | China | 1 | 0 | 0 | 0 | 0 |  |
| 74 | 28357098 | [Le Zhou](https://pubmed.ncbi.nlm.nih.gov/?term=Zhou+L&cauthor_id=28357098) | 2017 | China | 1 | 0 | 0 | 0 | 0 |  |
| 75 | 22490348 | [Dong Xie](https://pubmed.ncbi.nlm.nih.gov/?term=Xie+D&cauthor_id=22490348) | 2012 | China | 165 | 0 | 0 | 0 | 0 | Chinese |
| 76 | 23910931 | [Jiayu Zhang](https://pubmed.ncbi.nlm.nih.gov/?term=Zhang+J&cauthor_id=23910931) | 2014 | China | 18 | 0 | 0 | 0 | 0 |  |
| 77 | 25762452 | [G De Luca](https://pubmed.ncbi.nlm.nih.gov/?term=De+Luca+G&cauthor_id=25762452) | 2015 | Italy | 1 | 0 | 0 | 0 | 0 |  |
| 78 | 16861548 | [Myung Jin Chung](https://pubmed.ncbi.nlm.nih.gov/?term=Chung+MJ&cauthor_id=16861548) | 2006 | South Korea | 10 | 0 | 0 | 0 | 0 |  |
| 79 | 23290153 | [Aysegul Baysak](https://pubmed.ncbi.nlm.nih.gov/?term=Baysak+A&cauthor_id=23290153) | 2013 | Turkey | 1 | 0 | 0 | 0 | 0 |  |
| 80 | 26934286 | [Monica Onorati](https://pubmed.ncbi.nlm.nih.gov/?term=Onorati+M&cauthor_id=26934286) | 2016 | Italy | 1 | 0 | 0 | 0 | 0 |  |
| 81 | 16892484 | [Lian-Jin Jin](https://pubmed.ncbi.nlm.nih.gov/?term=Jin+LJ&cauthor_id=16892484) | 2006 | South Korea | 12 | 0 | 0 | 0 | 0 |  |
| 82 | 21467848 | [Ko-Han Lin](https://pubmed.ncbi.nlm.nih.gov/?term=Lin+KH&cauthor_id=21467848) | 2011 | Taiwan | 6 | 0 | 0 | 0 | 0 |  |
| 83 | 33145590 | Jennifer M Boland | 2021 | United States | 10 | 0 | 0 | 0 | 0 |  |
| 84 | 22263122 | [Joon Seok Park](https://pubmed.ncbi.nlm.nih.gov/?term=Park+JS&cauthor_id=22263122) | 2011 | South Korea | 32 | 0 | 0 | 0 | 0 |  |
| 85 | 26339444 | Hai-Miao Xu | 2015 | China | 1 | 1 | 0 | 0 | 0 |  |
| 86 | 21398141 | Amaravathi Sivaraman | 2011 | India | 1 | 0 | 0 | 0 | 0 |  |
| 87 | 25519861 | Keiji Yamanashi | 2014 | Japan | 1 | 0 | 0 | 0 | 0 |  |
| 88 | 19659837 | Po-Kuei Hsu | 2009 | Taipei | 2 | 0 | 0 | 0 | 0 |  |
| 89 | 22943472 | Chuang He | 2012 | China | 2 | 0 | 0 | 0 | 0 |  |
| 90 | 22614067 | Xu-Yong Lin | 2012 | China | 45 | 0 | 0 | 0 | 0 |  |
| 91 | 22291338 | Eugene Lee | 2013 | South Korea | 10 | 0 | 0 | 0 | 0 |  |
| 92 | 22394410 | Yoon-sung Bae | 2012 | South Korea | 1 | 0 | 1^6^ | 0 | 0 |  |
| 93 | 22740968 | Qingyong Chen | 2012 | China | 1 | 0 | 0 | 0 | 0 |  |
| 94 | 27812454 | Jin-Mei Wang | 2016 | China | 1 | 0 | 0 | 0 | 0 |  |
| 95 | 21859549 | Shaohua Ma | 2011 | China | 48 | 0 | 0 | 0 | 0 | Chinese |
| 96 | 18278694 | Wei S | 2008 | China | 1 | 0 | 0 | 1^7^ | 0 |  |
| 97 | 25210285 | [Koramadai Karuppusamy Kamaleshwaran](https://pubmed.ncbi.nlm.nih.gov/?term=Kamaleshwaran+KK&cauthor_id=25210285) | 2014 | India | 1 | 0 | 0 | 0 | 0 |  |
| 98 | 27909285 | [Kantaro Hara](https://pubmed.ncbi.nlm.nih.gov/?term=Hara+K&cauthor_id=27909285) | 2016 | Japan | 1 | 0 | 0 | 0 | 0 | Japanese |
| 99 | 20013629 | N Miura | 2009 | Japan | 1 | 0 | 0 | 0 | 0 |  |
| 100 | 21438340 | Soichi Oka | 2011 | Japan | 8 | 0 | 0 | 0 | 0 | Japanese |
| 101 | 22645055 | Andrew Dettrick | 2014 | Australia | 1 | 0 | 0 | 0 | 0 |  |
| 102 | 33816530 | [Gen Xu](https://pubmed.ncbi.nlm.nih.gov/?term=Xu+G&cauthor_id=33816530) | 2021 | China | 34 | 0 | 0 | 0 | 0 |  |
| 103 | 22883008 | Fei-yue Feng | 2012 | China | 89 | 0 | 0 | 0 | 0 | Chinese |
| 104 | 24887810 | [Tevfik Kaplan](https://pubmed.ncbi.nlm.nih.gov/?term=Kaplan+T&cauthor_id=24887810) | 2014 | Turkey | 1 | 0 | 0 | 0 | 0 |  |
| 105 | 12405907 | A C L Chan | 2002 | Hong Kong. | 20 | 0 | 0 | 0 | 0 |  |
| 106 | 29698656 | Juan A Santamaria-Barria | 2017 | United States | 1 | 0 | 0 | 0 | 0 |  |
| 107 | 17663895 | Félix Guerra-Gutiérrez | 2007 | Spain | 1 | 0 | 0 | 0 | 0 | Spanish |
| 108 | 27160435 | Chih-Ying Wu | 2016 | Taiwan | 14 | 0 | 0 | 0 | 0 |  |
| 109 | 23030396 | Baek-hui Kim | 2013 | South Korea | 29 | 1 | 0 | 0 | 0 |  |
| 110 | 21529795 | Q-B Wang | 2011 | China | 16 | 0 | 0 | 0 | 0 |  |
| 111 | 24322353 | Hidefumi Kita | 2013 | Japan | 1 | 1 | 0 | 0 | 0 | Japanese |
| 112 | 33725576 | Jefferson Chen | 2021 | United States | 1 | 0 | 0 | 0 | 0 |  |
| 113 | 15828298 | Jun Hanaoka | 2005 | Japan | 1 | 0 | 0 | 0 | 0 |  |
| 114 | 12887957 | Y-C Cheung | 2003 | Taiwan | 6 | 0 | 0 | 0 | 0 |  |
| 115 | 23227905 | Xu-Yong Lin | 2012 | China | 1 | 0 | 0 | 0 | 0 |  |
| 116 | 33660695 | Honghong Liu | 2021 | China | 11 | 0 | 0 | 0 | 0 |  |
| 117 | 16305908 | Hiromichi Katakura | 2005 | Japan | 1 | 1 | 0 | 0 | 0 |  |
| 118 | 30732701 | Avri Bohm | 2019 | United States | 1 | 0 | 0 | 0 | 0 |  |
| 119 | 22074388 | Giulio Rossi | 2012 | Italy | 10 | 0 | 0 | 0 | 0 |  |
| 120 | 23838116 | Nikolaos S Salemis | 2013 | Greece | 1 | 0 | 0 | 0 | 0 |  |
| 121 | 16753755 | Peter Robbins | 2006 | Australia | 1 | 0 | 0 | 0 | 0 |  |
| 122 | 19837274 | María J Pareja | 2009 | Spain | 1 | 0 | 0 | 0 | 0 |  |
| 123 | 1316026 | L T Chow | 1992 | Hong Kong | 1 | 0 | 0 | 0 | 0 |  |
| 124 | 15954523 | [Christoper M Stafford](https://pubmed.ncbi.nlm.nih.gov/?term=Stafford+CM&cauthor_id=15954523) | 2005 | United States | 1 | 0 | 0 | 0 | 0 |  |
| 125 | 23380035 | Domingo Ruiz de la Cuesta | 2013 | Spain | 1 | 0 | 0 | 0 | 0 | English and Spanish |
| 126 | 21645337 | Tobias S Schiergens | 2011 | Germany | 1 | 0 | 0 | 0 | 0 |  |
| 127 | 17383052 | Shun-Dong Dai | 2007 | China | 25 | 0 | 0 | 0 | 0 |  |
| 128 | 22691172 | Xu-Yong Lin | 2012 | China | 45 | 0 | 0 | 0 | 0 |  |
| 129 | 17473941 | Daan B de Koning | 2007 | Netherlands | 1 | 0 | 0 | 0 | 0 |  |
| 130 | 20732478 | Yunke Zhu | 2010 | China | 1 | 0 | 0 | 0 | 0 |  |
| 131 | 15121710 | [Y-Y Lu](https://pubmed.ncbi.nlm.nih.gov/?term=Lu+YY&cauthor_id=15121710) | 2004 | Taiwan | 1 | 0 | 0 | 0 | 0 |  |
| 132 | 14571291 | N G Chan | 2003 | Canada | 1 | 1 | 0 | 0 | 0 |  |
| 133 | 11075855 | A C Chan | 2000 | Hong Kong | 16 | 1 | 0 | 0 | 0 |  |
| 134 | 20634757 | Makiko Murota | 2011 | Japan | 1 | 0 | 0 | 0 | 0 |  |
| 135 | 17579131 | Hiroshi Takatani | 2007 | Japan | 1 | 0 | 0 | 0 | 0 |  |
| 136 | 29644341 | Georgios E Papadakis | 2018 | Switzerland | 1 | 0 | 0 | 0 | 0 |  |
| 137 | 21649526 | Hee Jin Lee | 2011 | South Korea | 24 | 2 | 1^8^ | 1 | 0 |  |
| 138 | 22454483 | Hiroaki Kuroda | 2012 | Japan | 1 | 0 | 0 | 0 | 0 |  |
| 139 | 3009921 | I Tanaka | 1986 | Japan | 1 | 1 | 0 | 0 | 0 |  |
| 140 | 12786904 | B M Majak | 2003 | Norway | 2 | 0 | 0 | 0 | 0 |  |
| 141 | 23345472 | Madhu Mati Goel | 2013 | India | 1 | 0 | 0 | 0 | 0 |  |
| 142 | 15188145 | Naoki Hosaka | 2004 | Japan | 1 | 0 | 0 | 0 | 0 |  |
| 143 | 32064104 | Han Luo | 2020 | China | 1 | 0 | 0 | 0 | 0 |  |
| 144 | 30702609 | Zhanlin Guo | 2004 | China | 21 | 0 | 0 | 0 | 0 | Chinese |
| 145 | 25022617 | Yihong Wang | 2014 | China | 1 | 0 | 0 | 0 | 0 |  |
| 146 | 12115302 | Akira Iyoda | 2002 | Japan | 15 | 0 | 0 | 0 | 0 |  |
| 147 | 33422233 | Nora Mayer | 2021 | Switzerland | 1 | 0 | 1^9^ | 0 | 0 |  |
| 148 | 15974816 | Seong Ho Yoo | 2005 | South Korea | 15 | 0 | 0 | 0 | 0 |  |
| 149 | 18710622 | Dong-Rong Situ | 2008 | China | 24 | 0 | 0 | 0 | 0 | Chinese |
| 150 | 21599956 | Wei Liu | 2011 | China | 1^10^ | 0 | 0 | 0 | 0 |  |
| 151 | 26223076 | S Van Petegem | 2015 | Belgium | 1 | 0 | 0 | 0 | 0 |  |
| 152 | 15511468 | Alberto M Marchevsky | 2004 | United States | 1 | 0 | 0 | 0 | 0 |  |
| 153 | 17873892 | En-Hua Wang | 2007 | China | 19 | 0 | 0 | 0 | 0 |  |
| 154 | 17685265 | A Pekcolaklar | 2007 | Turkey | 1 | 0 | 0 | 0 | 0 |  |
| 155 | 12878556 | Balakrishnan Mahesh | 2003 | UK | 3 | 0 | 0 | 0 | 0 |  |
| 156 | 20422318 | Li Gong | 2011 | China | 22 | 0 | 0 | 0 | 0 |  |
| 157 | 20715456 | Hisashi Oishi | 2010 | Japan | 7 | 0 | 0 | 0 | 0 | Japanese |
| 158 | 17914564 | [Su Jin Cho](https://pubmed.ncbi.nlm.nih.gov/?term=Cho+SJ&cauthor_id=17914564) | 2007 | South Korea | 3 | 0 | 0 | 0 | 0 |  |
| 159 | 33489116 | Albaraa Bara | 2021 | Syrian Arab Republic | 1 | 0 | 0 | 0 | 0 |  |
| 160 | 2540732 | [EM Nepomniashchaia](https://pubmed.ncbi.nlm.nih.gov/?term=Nepomniashchaia+EM&cauthor_id=2540732) | 1989 | Russia | 1 | 0 | 0 | 0 | 0 | Russian |
| 161 | 6291188 | Keeng-Wai Chan | 1982 | Hong Kong | 14 | 0 | 0 | 0 | 0 |  |
| 162 | 17693578 | P-M Chiang | 2008 | Taiwan | 37 | 0 | 0 | 0 | 0 |  |
| 163 | 17704262 | Y Wang | 2008 | China | 19 | 0 | 0 | 0 | 0 |  |
| 164 | 21881377 | Taichiro Goto | 2011 | Japan | 1 | 0 | 0 | 0 | 0 |  |
| 165 | 4331028 | [G Kaufman](https://pubmed.ncbi.nlm.nih.gov/?term=Kaufman+G&cauthor_id=4331028) | 1971 | United States | 1 | 0 | 0 | 0 | 0 |  |
| 166 | 16226111 | Chen-Tu Wu | 2005 | Taiwan | 37 | 0 | 0 | 0 | 0 |  |
| 167 | 19757636 | [Alberto Cavazza](https://pubmed.ncbi.nlm.nih.gov/?term=Cavazza+A&cauthor_id=19757636) | 2002 | Italy | 1 | 0 | 0 | 0 | 0 |  |
| 168 | 21144281 | Xiuwei Zhang | 2006 | China | 25 | 0 | 0 | 0 | 0 | Chinese |
| 169 | 15854960 | Fumihiro Tanaka | 2005 | Japan | 1 | 0 | 0 | 0 | 0 |  |
| 170 | 14636282 | Q van Wyk | 2003 | UK | 1 | 0 | 0 | 0 | 0 |  |
| 171 | 8702253 | Rizzo S | 1996 | Italy | 1 | 0 | 0 | 0 | 0 |  |
| 172 | 23756801 | G Rosliza | 2010 | Malaysia. | 1 | 0 | 0 | 0 | 0 |  |
| 173 | 12775260 | Ru-ming Xie | 2003 | China | 20 | 0 | 0 | 0 | 0 | Chinese |
| 174 | 10716159 | J Rodriguez-Soto | 2000 | United States | 21 | 0 | 0 | 0 | 0 |  |
| 175 | 28483135 | Sujith V Cherian | 2017 | United States | 1 | 0 | 0 | 0 | 0 |  |
| 176 | 7089615 | [L J Williams Jr](https://pubmed.ncbi.nlm.nih.gov/?term=Williams+LJ+Jr&cauthor_id=7089615) | 1982 | United States | 2 | 0 | 0 | 0 | 0 |  |
| 177 | 2257512 | [W B Dawson](https://pubmed.ncbi.nlm.nih.gov/?term=Dawson+WB&cauthor_id=2257512) | 1990 | Canada | 1 | 0 | 0 | 0 | 0 |  |
| 178 | 11892021 | [Anthony A Gal](https://pubmed.ncbi.nlm.nih.gov/?term=Gal+AA&cauthor_id=11892021) | 2002 | United States | 1 | 0 | 0 | 0 | 0 |  |
| 179 | 15585708 | Vyas J Soumil | 2004 | India | 1 | 0 | 0 | 0 | 0 |  |
| 180 | 22515818 | Yang Lei | 2012 | China | 28 | 0 | 0 | 0 | 0 |  |
| 181 | 33171926 | Jelena Stojšić | 2020 | Serbia | 1 | 0 | 0 | 0 | 0 |  |
| 182 | 15116333 | Enhua Wang | 2004 | China | 30 | 0 | 0 | 0 | 0 |  |
| 183 | 21710650 | Luis Z Blanco | 2013 | United States | 2 | 0 | 0 | 0 | 0 |  |
| 184 | 15681603 | Tomoyuki Hishida | 2005 | Japan | 1 | 0 | 0 | 0 | 0 |  |
| 185 | 23194051 | Lindsay A Schmidt | 2012 | United States | 6 | 0 | 0 | 0 | 0 |  |
| 186 | 23456164 | Michael Chu | 2013 | UK | 1 | 0 | 0 | 0 | 0 |  |
| 187 | 10895813 | M Devouassoux-Shisheboran | 2000 | United States | 100 | 1 | 0 | 0 | 0 |  |
| 188 | 27601661 | Seung-Hyun Jung | 2016 | South Korea | 43 | 0 | 0 | 0 | 0 |  |
| 189 | 12722330 | Sachie Tanno | 2003 | Japan | 1 | 0 | 0 | 0 | 0 | Japanese |
| 190 | 15270615 | Sanja Dacic | 2004 | United States | 9 | 0 | 0 | 0 | 0 |  |
| 191 | 15138814 | [Kazuto Yamazaki](https://pubmed.ncbi.nlm.nih.gov/?term=Yamazaki+K&cauthor_id=15138814) | 2004 | Japan | 6 | 0 | 0 | 0 | 0 |  |
| 192 | 21144300 | Fengjie Qi | 2006 | China | 19 | 0 | 0 | 0 | 0 | Chinese |
| 193 | 24518736 | [Kaushik Saha](https://pubmed.ncbi.nlm.nih.gov/?term=Saha+K&cauthor_id=24518736) | 2013 | India | 1 | 0 | 0 | 0 | 0 |  |
| 194 | 22832056 | [Yi Qian](https://pubmed.ncbi.nlm.nih.gov/?term=Qian+Y&cauthor_id=22832056) | 2012 | China | 1 | 0 | 0 | 0 | 0 |  |
| 195 | 12940776 | [J D Khoury](https://pubmed.ncbi.nlm.nih.gov/?term=Khoury+JD&cauthor_id=12940776) | 2003 | United States | 3 | 0 | 0 | 0 | 0 |  |
| 196 | 9754011 | [W Matsuyama](https://pubmed.ncbi.nlm.nih.gov/?term=Matsuyama+W&cauthor_id=9754011) | 1998 | Japan | 1 | 0 | 0 | 0 | 0 | Japanese |
| 197 | 1510507 | [R J Landreneau](https://pubmed.ncbi.nlm.nih.gov/?term=Landreneau+RJ&cauthor_id=1510507) | 1992 | United States | 1 | 0 | 0 | 0 | 0 |  |
| 198 | 7807757 | [Y Sagara](https://pubmed.ncbi.nlm.nih.gov/?term=Sagara+Y&cauthor_id=7807757) | 1994 | Japan | 1 | 0 | 0 | 0 | 0 | Japanese |
| 199 | 11747223 | [W K Ng](https://pubmed.ncbi.nlm.nih.gov/?term=Ng+WK&cauthor_id=11747223) | 2001 | Hong Kong | 1 | 0 | 0 | 0 | 0 |  |
| 200 | 21881332 | [Junji Ichinose](https://pubmed.ncbi.nlm.nih.gov/?term=Ichinose+J&cauthor_id=21881332) | 2011 | Japan | 1 | 0 | 0 | 0 | 0 |  |
| 201 | 25130377 | [Jian Wu](https://pubmed.ncbi.nlm.nih.gov/?term=Wu+J&cauthor_id=25130377) | 2014 | China | 18 | 0 | 0 | 0 | 0 |  |
| 202 | 17095978 | [Wolfgang Jungraithmayr](https://pubmed.ncbi.nlm.nih.gov/?term=Jungraithmayr+W&cauthor_id=17095978) | 2006 | Germany | 1 | 0 | 0 | 0 | 0 |  |
| 203 | 12405908 | [A G Nicholson](https://pubmed.ncbi.nlm.nih.gov/?term=Nicholson+AG&cauthor_id=12405908) | 2002 | UK | 3 | 1^11^ | 0 | 0 | 0 |  |
| 204 | 20013104 | [Ryo Maeda](https://pubmed.ncbi.nlm.nih.gov/?term=Maeda+R&cauthor_id=20013104) | 2009 | Japan | 1 | 0 | 0 | 0 | 0 |  |
| 205 | 12949064 | [Hiroshi Kitagawa](https://pubmed.ncbi.nlm.nih.gov/?term=Kitagawa+H&cauthor_id=12949064) | 2003 | Japan | 1 | 0 | 0 | 0 | 0 |  |
| 206 | 11955335 | [Jianqiang Zhang](https://pubmed.ncbi.nlm.nih.gov/?term=Zhang+J&cauthor_id=11955335) | 2002 | China | 17 | 0 | 0 | 0 | 0 | Chinese |
| 207 | 16484012 | [Hong Wui Tan](https://pubmed.ncbi.nlm.nih.gov/?term=Tan+HW&cauthor_id=16484012) | 2006 | Singapore | 1 | 0 | 0 | 0 | 0 |  |
| 208 | 26385193 | [Hideyuki Maeda](https://pubmed.ncbi.nlm.nih.gov/?term=Maeda+H&cauthor_id=26385193) | 2015 | Japan | 1 | 0 | 0 | 0 | 0 |  |
| 209 | 18844941 | [Keum H Choi](https://pubmed.ncbi.nlm.nih.gov/?term=Choi+KH&cauthor_id=18844941) | 2008 | South Korea | 1 | 0 | 0 | 0 | 0 |  |
| 210 | 17706129 | [Zhi-nong Jiang](https://pubmed.ncbi.nlm.nih.gov/?term=Jiang+ZN&cauthor_id=17706129) | 2007 | China | 1 | 1 | 0 | 0 | 0 | Chinese |
| 211 | 26728679 | [G Shao](https://pubmed.ncbi.nlm.nih.gov/?term=Shao+G&cauthor_id=26728679) | 2015 | China | 1 | 0 | 0 | 0 | 0 |  |
| 212 | 33782195 | [Zhanxian Shang](https://pubmed.ncbi.nlm.nih.gov/?term=Shang+Z&cauthor_id=33782195) | 2021 | China | 230 | 1 | 0 | 0 | 0 |  |
| 213 | 23168550 | [M Jawad Latif](https://pubmed.ncbi.nlm.nih.gov/?term=Latif+MJ&cauthor_id=23168550) | 2009 | United States | 1 | 0 | 0 | 0 | 0 |  |
| 214 | 16449928 | [B Smati](https://pubmed.ncbi.nlm.nih.gov/?term=Smati+B&cauthor_id=16449928) | 2005 | Tunisia | 1 | 0 | 0 | 0 | 0 | French |
| 215 | 2823596 | [N Nagata](https://pubmed.ncbi.nlm.nih.gov/?term=Nagata+N&cauthor_id=2823596) | 1987 | Japan | 16 | 0 | 0 | 0 | 0 |  |
| 216 | 27168758 | [Jennifer Zeng](https://pubmed.ncbi.nlm.nih.gov/?term=Zeng+J&cauthor_id=27168758) | 2016 | United States | 1 | 0 | 0 | 0 | 0 |  |
| 217 | 17242645 | [F Bougrine](https://pubmed.ncbi.nlm.nih.gov/?term=Bougrine+F&cauthor_id=17242645) | 2006 | Tunis | 1 | 0 | 0 | 0 | 0 | French |
| 218 | 16638358 | [Tao Wang](https://pubmed.ncbi.nlm.nih.gov/?term=Wang+T&cauthor_id=16638358) | 2006 | China | 1 | 0 | 0 | 0 | 0 | Chinese |
| 219 | 17895751 | [Giuliana Sartori](https://pubmed.ncbi.nlm.nih.gov/?term=Sartori+G&cauthor_id=17895751) | 2007 | Italy | 11 | 1 | 0 | 0 | 0 |  |
| 220 | 14704717 | [Mojgan Devouassoux-Shisheboran](https://pubmed.ncbi.nlm.nih.gov/?term=Devouassoux-Shisheboran+M&cauthor_id=14704717) | 2004 | France | 2 | 0 | 0 | 0 | 0 |  |
| 221 | 26193226 | [Mihai Danciu](https://pubmed.ncbi.nlm.nih.gov/?term=Danciu+M&cauthor_id=26193226) | 2015 | Romania | 1 | 0 | 0 | 0 | 0 |  |
| 222 | 33495182 | [Sven L Van Laer](https://pubmed.ncbi.nlm.nih.gov/?term=Van+Laer+SL&cauthor_id=33495182) | 2021 | Belgium | 1 | 0 | 0 | 0 | 0 |  |
| 223 | 18245708 | [Mohamed-Sadok Boudaya](https://pubmed.ncbi.nlm.nih.gov/?term=Boudaya+MS&cauthor_id=18245708) | 2008 | France | 1 | 0 | 0 | 0 | 0 |  |
| 224 | 16856537 | [K Suzuki](https://pubmed.ncbi.nlm.nih.gov/?term=Suzuki+K&cauthor_id=16856537) | 2006 | Japan | 1 | 0 | 0 | 0 | 0 | Japanese |
| 225 | 11570910 | [P B Illei](https://pubmed.ncbi.nlm.nih.gov/?term=Illei+PB&cauthor_id=11570910) | 2001 | United States | 9 | 0 | 0 | 0 | 0 |  |
| 226 | 18363024 | [José Pedro Boléo-Tomé](https://pubmed.ncbi.nlm.nih.gov/?term=Pedro+Bol%C3%A9o-Tom%C3%A9+J&cauthor_id=18363024) | 2008 | Portugal | 1 | 0 | 0 | 0 | 0 | Portuguese |
| 227 | 11899220 | [Motoki Yano](https://pubmed.ncbi.nlm.nih.gov/?term=Yano+M&cauthor_id=11899220) | 2002 | Japan | 1 | 1 | 0 | 0 | 0 |  |
| 228 | 20387533 | [Eiichiro Anan](https://pubmed.ncbi.nlm.nih.gov/?term=Anan+E&cauthor_id=20387533) | 2010 | Japan | 1 | 1 | 0 | 0 | 0 | Japanese |
| 229 | 7286919 | [E Alvarez-Fernandez](https://pubmed.ncbi.nlm.nih.gov/?term=Alvarez-Fernandez+E&cauthor_id=7286919) | 1981 | Spain | 1 | 0 | 0 | 0 | 0 |  |
| 230 | 9192438 | [K Nakanishi](https://pubmed.ncbi.nlm.nih.gov/?term=Nakanishi+K&cauthor_id=9192438) | 1997 | Japan | 1 | 0 | 0 | 0 | 0 |  |
| 231 | 14720435 | [Xiang-hua Yi](https://pubmed.ncbi.nlm.nih.gov/?term=Yi+XH&cauthor_id=14720435) | 2003 | China | 42 | 0 | 0 | 0 | 0 | Chinese |
| 232 | 18067639 | [Randa M S Amin](https://pubmed.ncbi.nlm.nih.gov/?term=Amin+RM&cauthor_id=18067639) | 2008 | Japan | 6 | 0 | 0 | 0 | 0 |  |
| 233 | 1515703 | [M Barbareschi](https://pubmed.ncbi.nlm.nih.gov/?term=Barbareschi+M&cauthor_id=1515703) | 1992 | Italy | 1 | 0 | 0 | 0 | 0 |  |
| 234 | 4342853 | [G S Hill](https://pubmed.ncbi.nlm.nih.gov/?term=Hill+GS&cauthor_id=4342853) | 1972 | United States | 1 | 0 | 0 | 0 | 0 |  |
| 235 | 24238520 | [Hui Zhao](https://pubmed.ncbi.nlm.nih.gov/?term=Zhao+H&cauthor_id=24238520) | 2013 | China | 2 | 0 | 0 | 0 | 0 |  |
| 236 | 17525638 | [Eugenio Leonardo](https://pubmed.ncbi.nlm.nih.gov/?term=Leonardo+E&cauthor_id=17525638) | 2007 | Italy | 8 | 0 | 0 | 0 | 0 |  |
| 237 | 8282879 | [J G Im](https://pubmed.ncbi.nlm.nih.gov/?term=Im+JG&cauthor_id=8282879) | 1994 | South Korea | 8 | 0 | 0 | 0 | 0 |  |
| 238 | 15176609 | [Ikuko Sakamoto](https://pubmed.ncbi.nlm.nih.gov/?term=Sakamoto+I&cauthor_id=15176609) | 2004 | Japan | 1 | 0 | 0 | 0 | 0 |  |
| 239 | 12016362 | [Ji Eun Nam](https://pubmed.ncbi.nlm.nih.gov/?term=Nam+JE&cauthor_id=12016362) | 2002 | South Korea | 2 | 0 | 0 | 0 | 0 |  |
| 240 | 8821986 | [W Chiba](https://pubmed.ncbi.nlm.nih.gov/?term=Chiba+W&cauthor_id=8821986) | 1995 | Japan | 8 | 0 | 0 | 0 | 0 | Japanese |
| 241 | 12619190 | [Kyung-Hee Kim](https://pubmed.ncbi.nlm.nih.gov/?term=Kim+KH&cauthor_id=12619190) | 2003 | South Korea | 1 | 1 | 0 | 0 | 0 |  |
| 242 | 22182444 | [Zhongmin Peng](https://pubmed.ncbi.nlm.nih.gov/?term=Peng+Z&cauthor_id=22182444) | 2012 | China | 4 | 0 | 0 | 0 | 0 |  |
| 243 | 2981138 | [N Nagata](https://pubmed.ncbi.nlm.nih.gov/?term=Nagata+N&cauthor_id=2981138) | 1985 | Japan | 13 | 0 | 0 | 0 | 0 |  |
| 244 | 17144585 | [Kazuhisa Ito](https://pubmed.ncbi.nlm.nih.gov/?term=Ito+K&cauthor_id=17144585) | 2006 | Japan | 1 | 0 | 0 | 0 | 0 | Japanese |
| 245 | 17120028 | [Yoji Wani](https://pubmed.ncbi.nlm.nih.gov/?term=Wani+Y&cauthor_id=17120028) | 2007 | Japan | 1 | 0 | 0 | 0 | 0 |  |
| 246 | 23157959 | [Qiang Pu](https://pubmed.ncbi.nlm.nih.gov/?term=Pu+Q&cauthor_id=23157959) | 2012 | China | 1 | 0 | 0 | 0 | 0 | Chinese |
| 247 | 21248463 | [Ahmet Celik](https://pubmed.ncbi.nlm.nih.gov/?term=Celik+A&cauthor_id=21248463) | 200 | Turkey | 1 | 0 | 0 | 0 | 0 |  |
| 248 | 20629338 | [Qiang Pu](https://pubmed.ncbi.nlm.nih.gov/?term=Pu+Q&cauthor_id=20629338) | 2010 | China | 6 | 0 | 0 | 0 | 0 | Chinese |
| 249 | 16776996 | [Feng-jie Qi](https://pubmed.ncbi.nlm.nih.gov/?term=Qi+FJ&cauthor_id=16776996) | 2006 | China | 17 | 0 | 0 | 0 | 0 | Chinese |
| 250 | 33884249 | [Mohamed Abdelghaffar](https://pubmed.ncbi.nlm.nih.gov/?term=Abdelghaffar+M&cauthor_id=33884249) | 2021 | Bahrain | 1^12^ | 0 | 0 | 0 | 0 |  |
| 251 | 23894794 | [Anca Macri](https://pubmed.ncbi.nlm.nih.gov/?term=Macri+A&cauthor_id=23894794) | 2013 | Romania | 1 | 0 | 0 | 0 | 0 | Romanian |
| 252 | 18443540 | [K Murdzhev](https://pubmed.ncbi.nlm.nih.gov/?term=Murdzhev+K&cauthor_id=18443540) | 2007 | Bulgaria | 1 | 0 | 0 | 0 | 0 | Bulgarian |
| 253 | 2537067 | [E Alvarez-Fernandez](https://pubmed.ncbi.nlm.nih.gov/?term=Alvarez-Fernandez+E&cauthor_id=2537067) | 1989 | Spain | 3 | 0 | 0 | 0 | 0 |  |
| 254 | 1766156 | [K Murakami](https://pubmed.ncbi.nlm.nih.gov/?term=Murakami+K&cauthor_id=1766156) | 1991 | Japan | 2 | 0 | 0 | 0 | 0 | Japanese |
| 255 | 15379333 | [Gonzalo Cardemil](https://pubmed.ncbi.nlm.nih.gov/?term=Cardemil+G&cauthor_id=15379333) | 2004 | Chile | 1 | 0 | 0 | 0 | 0 | Spanish |
| 256 | 12408771 | [Dongmei Lin](https://pubmed.ncbi.nlm.nih.gov/?term=Lin+D&cauthor_id=12408771) | 2002 | China | 36 | 0 | 0 | 0 | 0 |  |
| 257 | 15084379 | [Yih-Leong Chang](https://pubmed.ncbi.nlm.nih.gov/?term=Chang+YL&cauthor_id=15084379) | 2004 | Taiwan | 44 | 0 | 0 | 0 | 0 |  |
| 258 | 16206654 | [Raffaele Longo](https://pubmed.ncbi.nlm.nih.gov/?term=Longo+R&cauthor_id=16206654) | 2005 | Italy | 1 | 0 | 0 | 0 | 0 |  |
| 259 | 19365977 | [Shahidul Islam](https://pubmed.ncbi.nlm.nih.gov/?term=Islam+S&cauthor_id=19365977) | 2009 | Canada | 1 | 0 | 0 | 0 | 0 |  |
| 260 | 1716416 | [N P Ohori](https://pubmed.ncbi.nlm.nih.gov/?term=Ohori+NP&cauthor_id=1716416) | 1991 | United States | 4 | 0 | 0 | 0 | 0 |  |
| 261 | 21057447 | [Takeshi Mori](https://pubmed.ncbi.nlm.nih.gov/?term=Mori+T&cauthor_id=21057447) | 2010 | Japan | 1 | 0 | 0 | 0 | 0 |  |
| 262 | 8697515 | [Z Li](https://pubmed.ncbi.nlm.nih.gov/?term=Li+Z&cauthor_id=8697515) | 1995 | China | 11 | 0 | 0 | 0 | 0 | Chinese |
| 263 | 8549174 | [A P Yim](https://pubmed.ncbi.nlm.nih.gov/?term=Yim+AP&cauthor_id=8549174) | 1996 | Hong Kong | 1 | 0 | 0 | 0 | 0 |  |
| 264 | 7944888 | [C A Moran](https://pubmed.ncbi.nlm.nih.gov/?term=Moran+CA&cauthor_id=7944888) | 1994 | United States | 1 | 0 | 0 | 0 | 0 |  |
| 265 | 6307535 | [P Mirejovský](https://pubmed.ncbi.nlm.nih.gov/?term=Mirejovsk%C3%BD+P&cauthor_id=6307535) | 1983 | Czech Republic | 1 | 0 | 0 | 0 | 0 | Czech |
| 266 | 8082241 | [W H Li](https://pubmed.ncbi.nlm.nih.gov/?term=Li+WH&cauthor_id=8082241) | 1994 | China | 31 | 0 | 0 | 0 | 0 | Chinese |
| 267 | 3021315 | [M Huszar](https://pubmed.ncbi.nlm.nih.gov/?term=Huszar+M&cauthor_id=3021315) | 1986 | Israel | 1 | 0 | 0 | 0 | 0 |  |
| 268 | 213375 | [E Heilman](https://pubmed.ncbi.nlm.nih.gov/?term=Heilman+E&cauthor_id=213375) | 1978 | United States | 1 | 0 | 0 | 0 | 0 |  |
| 269 | 2560946 | [B Bednár](https://pubmed.ncbi.nlm.nih.gov/?term=Bedn%C3%A1r+B&cauthor_id=2560946) | 1989 | Czech Republic | 1 | 0 | 0 | 0 | 0 | Czech |
| 270 | 12222919 | [Akihiro Hayashi](https://pubmed.ncbi.nlm.nih.gov/?term=Hayashi+A&cauthor_id=12222919) | 2002 | Japan | 1 | 0 | 0 | 0 | 0 |  |
| 271 | 8058573 | [P Heikkilä](https://pubmed.ncbi.nlm.nih.gov/?term=Heikkil%C3%A4+P&cauthor_id=8058573) | 1994 | Finland | 2 | 0 | 0 | 0 | 0 |  |
| 272 | 2375219 | [C Anderson](https://pubmed.ncbi.nlm.nih.gov/?term=Anderson+C&cauthor_id=2375219) | 1990 | United States | 1 | 0 | 0 | 0 | 0 |  |
| 273 | 3013747 | [H Spencer](https://pubmed.ncbi.nlm.nih.gov/?term=Spencer+H&cauthor_id=3013747) | 1986 | Japan | 29 | 1 | 0 | 0 | 0 |  |
| 274 | 19402838 | [Nai-Chuan Chien](https://pubmed.ncbi.nlm.nih.gov/?term=Chien+NC&cauthor_id=19402838) | 2009 | Taiwan | 1 | 1 |  |  |  |  |
| 275 | 15201499 | Gou Young Kim | 2004 | South Korea | 16 | 1 | 0 | 0 | 0 |  |
| 276 | 19679971 | [Pradeep Vaideeswar](https://pubmed.ncbi.nlm.nih.gov/?term=Vaideeswar+P&cauthor_id=19679971) | 2009 | India | 1 | 1 | 0 | 0 | 0 |  |
| 277 | 1309991 | K Sugio | 1992 | Japan | 10 | 0 | 0 | 0 | 0 |  |
| 278 | 12728972 | [Kuang-Tai Kuo](https://pubmed.ncbi.nlm.nih.gov/?term=Kuo+KT&cauthor_id=12728972) | 2003 | Taiwan | 44 | 0 | 0 | 0 | 0 |  |
| 279 | 225006 | [J J Palacios](https://pubmed.ncbi.nlm.nih.gov/?term=Palacios+JJ&cauthor_id=225006) | 1979 | Spain | 1 | 0 | 0 | 0 | 0 |  |
| 280 | 196998 | S Kay | 1977 | United States | 1 | 0 | 0 | 0 | 0 |  |
| 281 | 27222778 | [Davide Patrini](https://pubmed.ncbi.nlm.nih.gov/?term=Patrini+D&cauthor_id=27222778) | 2015 | UK | 1 | 0 | 0 | 0 | 0 |  |
| 282 | 9298877 | H M xu | 1997 | China | 32 | 0 | 0 | 0 | 0 |  |
| 283 | 6252791 | A L Katzenstein | 1980 | United States | 51 | 0 | 0 | 0 | 0 |  |
| 284 | 3717498 | [M Noguchi](https://pubmed.ncbi.nlm.nih.gov/?term=Noguchi+M&cauthor_id=3717498) | 1986 | Japan | 1 | 0 | 0 | 0 | 0 |  |
| 285 | 13284701 | A A LIEBOW | 1956 | United States | 7 | 0 | 0 | 0 | 0 |  |
| 286 | 9546367 | S Niho | 1998 | Japan | 6 | 0 | 0 | 0 | 0 |  |
| 287 | 8237370 | [T Aihara](https://pubmed.ncbi.nlm.nih.gov/?term=Aihara+T&cauthor_id=8237370) | 1993 | Japan | 13 | 0 | 0 | 0 | 0 |  |
| 288 | 12645743 | [Kazuhiro Sakamoto](https://pubmed.ncbi.nlm.nih.gov/?term=Sakamoto+K&cauthor_id=12645743) | 2003 | Japan | 1 | 0 | 0 | 0 | 0 |  |
| 289 | 4340663 | J E Haas | 1972 | United States | 1 | 0 | 0 | 0 | 0 |  |
| 290 | 12166263 | [Etsu Tsuduki](https://pubmed.ncbi.nlm.nih.gov/?term=Tsuduki+E&cauthor_id=12166263) | 2002 | Japan | 1 | 0 | 0 | 0 | 0 | Japanese |
| 291 | 2545966 | [K Maezato](https://pubmed.ncbi.nlm.nih.gov/?term=Maezato+K&cauthor_id=2545966) | 1989 | Japan | 1 | 0 | 0 | 0 | 0 | Japanese |
| 292 | 8958724 | [M Nomura](https://pubmed.ncbi.nlm.nih.gov/?term=Nomura+M&cauthor_id=8958724) | 1996 | Japan | 1 | 0 | 0 | 0 | 0 | Japanese |
| 293 | 21144281 | Xiuwei Zhang | 2006 | China | 25 | 0 | 0 | 0 | 0 | Chinese |
| 294 | 21262155 | [Yan Wang](https://pubmed.ncbi.nlm.nih.gov/?term=Wang+Y&cauthor_id=21262155) | 2003 | China | 30 | 0 | 0 | 0 | 0 | Chinese |
| 295 | 8249516 | [Y T Kaw](https://pubmed.ncbi.nlm.nih.gov/?term=Kaw+YT&cauthor_id=8249516) | 1993 | United States | 1 | 0 | 0 | 0 | 0 |  |
| 296 | 7667514 | [F Fujiyoshi](https://pubmed.ncbi.nlm.nih.gov/?term=Fujiyoshi+F&cauthor_id=7667514) | 1995 | Japan | 1 | 0 | 0 | 0 | 0 |  |
| 297 | 208101 | [Y W Bahk](https://pubmed.ncbi.nlm.nih.gov/?term=Bahk+YW&cauthor_id=208101) | 1978 | South Korea | 2 | 0 | 0 | 0 | 0 |  |
| 298 | 9843249 | [F Fujiyoshi](https://pubmed.ncbi.nlm.nih.gov/?term=Fujiyoshi+F&cauthor_id=9843249) | 1998 | Japan | 2 | 0 | 0 | 0 | 0 |  |
| 299 | 12938610 | [Nermin Halkic](https://pubmed.ncbi.nlm.nih.gov/?term=Halkic+N&cauthor_id=12938610) | 2003 | Switzerland | 1 | 0 | 0 | 0 | 0 |  |
| 300 | 2847478 | [M Aiba](https://pubmed.ncbi.nlm.nih.gov/?term=Aiba+M&cauthor_id=2847478) | 1988 | Japan | 1 | 0 | 0 | 0 | 0 |  |
| 301 | 2463730 | M Fukayama | 1988 | Japan | 3 | 0 | 0 | 0 | 0 |  |
| 302 | 6187231 | [A L Katzenstein](https://pubmed.ncbi.nlm.nih.gov/?term=Katzenstein+AL&cauthor_id=6187231) | 1983 | United States | 9 | 0 | 0 | 0 | 0 |  |
| 303 | **8577531** | [L Guibaud](https://pubmed.ncbi.nlm.nih.gov/?term=Guibaud+L&cauthor_id=8577531) | 1995 | France | 1 | 0 | 0 | 0 | 0 |  |
| 304 | 5676973 | [S Mori](https://pubmed.ncbi.nlm.nih.gov/?term=Mori+S&cauthor_id=5676973) | 1968 | Japan | 1 | 0 | 0 | 0 | 0 |  |
| 305 | 2173679 | T Mitsudomi | 1990 | Japan | 6 | 0 | 0 | 0 | 0 |  |
| 306 | 11089322 | [R C Gupta](https://pubmed.ncbi.nlm.nih.gov/?term=Gupta+RC&cauthor_id=11089322) | 2000 | India | 1 | 0 | 0 | 0 | 0 |  |
| 307 | 8395613 | Y Matsuki | 1993 | Japan | 2 | 0 | 0 | 0 | 0 | Japanese |
| 308 | - 8684643 | [M Mezzetti](https://pubmed.ncbi.nlm.nih.gov/?term=Mezzetti+M&cauthor_id=8684643) | 1995 | Italy | 1 | 0 | 0 | 0 | 0 | Italian |
| 309 | 2164378 | [S G Kireeva](https://pubmed.ncbi.nlm.nih.gov/?term=Kireeva+SG&cauthor_id=2164378) | 1990 | Russia | 1 | 0 | 0 | 0 | 0 | Russian |
| 310 | 8956547 | [M R Ballotta](https://pubmed.ncbi.nlm.nih.gov/?term=Ballotta+MR&cauthor_id=8956547) | 1996 | Italy | 1 | 0 | 0 | 0 | 0 |  |
| 311 | 11550723 | [M Hara](https://pubmed.ncbi.nlm.nih.gov/?term=Hara+M&cauthor_id=11550723) | 2001 | Japan | 1 | 0 | 0 | 0 | 0 |  |
| 312 | 8620735 | A P Yim | 1996 | Hong Kong | 1 | 0 | 0 | 0 | 0 |  |
| 313 | 12715177 | [Rie Shibata](https://pubmed.ncbi.nlm.nih.gov/?term=Shibata+R&cauthor_id=12715177) | 2003 | Japan | 1 | 0 | 0 | 0 | 0 |  |
| 314 | 7745988 | [M B Majak](https://pubmed.ncbi.nlm.nih.gov/?term=Majak+MB&cauthor_id=7745988) | 1994 | Norway | 1 | 0 | 0 | 0 | 0 |  |
| 315 | **8519196** | [E M Wojcik](https://pubmed.ncbi.nlm.nih.gov/?term=Wojcik+EM&cauthor_id=8519196) | 1993 | United States | 1 | 0 | 0 | 0 | 0 |  |
| 316 | 13561238 | E H RUBIN | 1958 | United States | 2 | 0 | 0 | 0 | 0 |  |
| 317 | 13862139 | [V M AREAN](https://pubmed.ncbi.nlm.nih.gov/?term=AREAN+VM&cauthor_id=13862139) | 1962 | United States | 1 | 0 | 0 | 0 | 0 |  |
| 318 | **33717587** | Chao Zhou | 2021 | China | 2 | 0 | 0 | 0 | 0 |  |
| 319 | 19026830 | [Jung-Jyh Hung](https://pubmed.ncbi.nlm.nih.gov/?term=Hung+JJ&cauthor_id=19026830) | 2008 | Taiwan | 1 | 0 | 0 | 0 | 0 |  |
| 320 | - **11895496** | [H Hattori](https://pubmed.ncbi.nlm.nih.gov/?term=Hattori+H&cauthor_id=11895496) | 2002 | Japan | 5 | 0 | 0 | 0 | 0 |  |
| 321 | 30488193 | [Masaki Ikeda](https://pubmed.ncbi.nlm.nih.gov/?term=Ikeda+M&cauthor_id=30488193) | 2019 | Japan | 1 | 0 | 0 | 0 | 0 |  |
| 322 | - **8835260** | [A S Leong](https://pubmed.ncbi.nlm.nih.gov/?term=Leong+AS&cauthor_id=8835260) | 1995 | Australia | 25 | 0 | 0 | 0 | 0 |  |
| 323 | **23773456** | [Young Kim](https://pubmed.ncbi.nlm.nih.gov/?term=Kim+Y&cauthor_id=23773456) | 2013 | South Korea | 1^13^ | 0 | 0 | 0 | 0 |  |
| 324 | **2167037** | [U S Salminen](https://pubmed.ncbi.nlm.nih.gov/?term=Salminen+US&cauthor_id=2167037) | 1990 | Finland | 4 | 0 | 0 | 0 | 0 |  |
| 325 | 6247707 | [K Joshi](https://pubmed.ncbi.nlm.nih.gov/?term=Joshi+K&cauthor_id=6247707) | 1980 | India | 1 | 0 | 0 | 0 | 0 |  |
| 326 | 2561731 | [Y K Park](https://pubmed.ncbi.nlm.nih.gov/?term=Park+YK&cauthor_id=2561731) | 1989 | South Korea | 2 | 0 | 0 | 0 | 0 |  |
| 327 | 4356266 | [A Kennedy](https://pubmed.ncbi.nlm.nih.gov/?term=Kennedy+A&cauthor_id=4356266) | 1973 | UK | 2 | 0 | 0 | 0 | 0 |  |
| 328 | **12101502** | [S Batinica](https://pubmed.ncbi.nlm.nih.gov/?term=Batinica+S&cauthor_id=12101502) | 2002 | Croatia | 1 | 0 | 0 | 0 | 0 |  |
| 329 | 8553108 | [C K Liam](https://pubmed.ncbi.nlm.nih.gov/?term=Liam+CK&cauthor_id=8553108) | 1995 | Malaysia | 1 | 0 | 0 | 0 | 0 |  |
| 330 | - 1310458 | [S T Lee](https://pubmed.ncbi.nlm.nih.gov/?term=Lee+ST&cauthor_id=1310458) | 1992 | China | 1 | 0 | 0 | 0 | 0 |  |
| 331 | - 8129367 | [P Y Cheong](https://pubmed.ncbi.nlm.nih.gov/?term=Cheong+PY&cauthor_id=8129367) | 1993 | Singapore | 1 | 0 | 0 | 0 | 0 |  |
| 332 | - 12811433 | [Ali Ahmetoğlu](https://pubmed.ncbi.nlm.nih.gov/?term=Ahmeto%C4%9Flu+A&cauthor_id=12811433) | 2003 | Turkey | 1 | 0 | 0 | 0 | 0 |  |
| 333 | - 9893427 | [H Takahashi](https://pubmed.ncbi.nlm.nih.gov/?term=Takahashi+H&cauthor_id=9893427) | 1998 | Japan | 1 | 0 | 0 | 0 | 0 | Japanese |
| 334 | - 191138 | [W R Webb](https://pubmed.ncbi.nlm.nih.gov/?term=Webb+WR&cauthor_id=191138) | 1977 | United States | 1 | 0 | 0 | 0 | 0 |  |
| 335 | - 8203922 | [W Chiba](https://pubmed.ncbi.nlm.nih.gov/?term=Chiba+W&cauthor_id=8203922) | 1994 | Japan | 1 | 0 | 0 | 0 | 0 | Japanese |
| 336 | - 2840832 | [S A Yousem](https://pubmed.ncbi.nlm.nih.gov/?term=Yousem+SA&cauthor_id=2840832) | 1988 | United States | 8 | 0 | 0 | 0 | 0 |  |
| 337 | - 7795162 | [F Sant](https://pubmed.ncbi.nlm.nih.gov/?term=Sant+F&cauthor_id=7795162) | 1995 | Spain | 1 | 0 | 0 | 0 | 0 |  |
| 338 | - 6318684 | [G Singh](https://pubmed.ncbi.nlm.nih.gov/?term=Singh+G&cauthor_id=6318684) | 1984 | United States | 2 | 0 | 0 | 0 | 0 |  |
| 339 | - 8210620 | [H Hirano](https://pubmed.ncbi.nlm.nih.gov/?term=Hirano+H&cauthor_id=8210620) | 1993 | Japan | 1 | 0 | 0 | 0 | 0 |  |
| 340 | - 3010798 | [A Thomas](https://pubmed.ncbi.nlm.nih.gov/?term=Thomas+A&cauthor_id=3010798) | 1986 | Singapore | 11 | 0 | 0 | 0 | 0 |  |
| 341 | - 18983474 | [R Saluja](https://pubmed.ncbi.nlm.nih.gov/?term=Saluja+R&cauthor_id=18983474) | 2008 | UK | 2 | 0 | 0 | 0 | 0 |  |
| 342 | - 27407347 | [SC TEWARI](https://www.ncbi.nlm.nih.gov/pubmed/?term=TEWARI%20S%5BAuthor%5D&cauthor=true&cauthor_uid=27407347) | 2001 | India | 1 | 0 | 0 | 0 | 0 |  |
| 343 | - 20675249 | [Nilendu C Purandare](https://pubmed.ncbi.nlm.nih.gov/?term=Purandare+NC&cauthor_id=20675249) | 2010 | India | 1 | 0 | 0 | 0 | 0 |  |
| 344 | - 12227952 | [Douglas A Weeks](https://pubmed.ncbi.nlm.nih.gov/?term=Weeks+DA&cauthor_id=12227952) | 2002 | United States | 1 | 0 | 0 | 0 | 0 |  |
| 345 | - 8277635 | [H Nakahashi](https://pubmed.ncbi.nlm.nih.gov/?term=Nakahashi+H&cauthor_id=8277635) | 1994 | Japan | 8 | 0 | 0 | 0 | 0 | Japanese |
| 346 | - 31020057 | [Estela Benito-Martínez](https://pubmed.ncbi.nlm.nih.gov/?term=Benito-Mart%C3%ADnez+E&cauthor_id=31020057) | 2019 | Spain | 1 | 0 | 0 | 0 | 0 |  |
| 347 | - 2413615 | [H Haimoto](https://pubmed.ncbi.nlm.nih.gov/?term=Haimoto+H&cauthor_id=2413615) | 1985 | Japan | 7 | 0 | 0 | 0 | 0 |  |
| 348 | - 21251411 | [Zhanlin Guo](https://pubmed.ncbi.nlm.nih.gov/?term=Guo+Z&cauthor_id=21251411) | 2004 | China | 21 | 0 | 0 | 0 | 0 | Chinese |
| 349 | - 1243931 | [Young Cheol Yoon](https://pubmed.ncbi.nlm.nih.gov/?term=Yoon+YC&cauthor_id=12439315) | 2002 | South Korea | 1 | 0 | 0 | 0 | 0 |  |
| 350 | - 16620664 | [Chang-hai Yu](https://pubmed.ncbi.nlm.nih.gov/?term=Yu+CH&cauthor_id=16620664) | 2006 | China | 2 | 0 | 0 | 0 | 0 | Chinese |
| 351 | - 17045865 | [Marco Sperandeo](https://pubmed.ncbi.nlm.nih.gov/?term=Sperandeo+M&cauthor_id=17045865) | 2006 | Italy | 1 | 0 | 0 | 0 | 0 |  |
| 352 | - 16292630 | [Mariëtte C A van Kouwen](https://pubmed.ncbi.nlm.nih.gov/?term=van+Kouwen+MC&cauthor_id=16292630) | 2005 | Netherlands | 1 | 0 | 0 | 0 | 0 |  |
| 353 | - 32792168 | [Jordi Juanola-Pla](https://pubmed.ncbi.nlm.nih.gov/?term=Juanola-Pla+J&cauthor_id=32792168) | 2021 | Spain | 1 | 0 | 0 | 0 | 0 | English and Spanish |
| 354 | - 11097406 | [F Li](https://pubmed.ncbi.nlm.nih.gov/?term=Li+F&cauthor_id=11097406) | 2000 | Japan | 1 | 0 | 0 | 0 | 0 |  |
| 355 | 34485321 | [Weidong Zhang](https://www.ncbi.nlm.nih.gov/pubmed/?term=Zhang%20W%5BAuthor%5D&cauthor=true&cauthor_uid=34485321) | 2021 | China | 1 | 1 | 1^14^ | 0 | 1^15^ |  |

1 Only non-English articles are displayed in the language column

2 Recurrence after segmentectomy at the same location of the left lower lobe

3 Local lymph node recurrence at station 9, 11, and 12

4 Pleural Dissemination

5 Bone metastases

6 Stomach

7 Recurrence after wedge resection at the same location of the left lower lobe

8 Lumbar spinal metastases

9 Pleural metastasized typical carcinoids mixed with pulmonary sclerosing pneumocytoma (PSP)

10 Coexistence of PSP and primary adenocarcinoma in the same nodule of lung

11 PSP combined with typical carcinoid, in which there was a mediastinal lymph node metastasis solely comprising the solid component of PSP

12 Coexistence of PSP and pulmonary hamartoma

13 First case of multiple peripheral typical carcinoid tumors associated with PSP

14 Liver, abdominal cavity, bones

15 The first patient to die of respiratory and circulatory failure caused by the PSP tumor and metastases compressing the mediastinal tissue
